# Strict gut symbiont specificity in Coreoidea insects governed by interspecies competition within *Caballeronia* strains

**DOI:** 10.1093/ismejo/wraf240

**Published:** 2025-11-13

**Authors:** Gaëlle Lextrait, Srotoswini Joardar, Raynald Cossard, Yoshitomo Kikuchi, Tsubasa Ohbayashi, Peter Mergaert

**Affiliations:** Université Paris-Saclay, CEA, CNRS, Institute for Integrative Biology of the Cell (I2BC), 91198 Gif-sur-Yvette, France; Université Paris-Saclay, CEA, CNRS, Institute for Integrative Biology of the Cell (I2BC), 91198 Gif-sur-Yvette, France; Department of Biology, Ludwig-Maximilians-Universität München (LMU), 82152 Munich, Germany; Comparative Microbiome Analysis (COMI), Helmholtz Zentrum München, 85764 Oberschleissheim, Germany; Université Paris-Saclay, CEA, CNRS, Institute for Integrative Biology of the Cell (I2BC), 91198 Gif-sur-Yvette, France; Bioproduction Research Institute, National Institute of Advanced Industrial Science and Technology (AIST), Hokkaido Center, 062-8517 Sapporo, Japan; Université Paris-Saclay, CEA, CNRS, Institute for Integrative Biology of the Cell (I2BC), 91198 Gif-sur-Yvette, France; Institute for Agro-Environmental Sciences, National Agriculture, and Food Research Organization (NARO), 305-8604 Tsukuba, Japan; Université Paris-Saclay, CEA, CNRS, Institute for Integrative Biology of the Cell (I2BC), 91198 Gif-sur-Yvette, France

**Keywords:** bacteria-bacteria competition, insect-bacteria interactions, gut symbiosis, *Riptortus pedestris*, *Coreus marginatus*, *Caballeronia*, chemotaxis, antimicrobial peptides, metabolic capacity

## Abstract

Host-bacteria symbioses are specific and transgenerationaly stable. In hosts that acquire their symbionts from the environment, selective mechanisms are required to identify beneficial partners among environmental microorganisms. In Coreoidea stinkbugs, which house environmentally acquired symbionts in the midgut, bacterial competition shapes symbiont specificity whereby *Caballeronia* strains consistently outcompete other bacteria. Here, we show that competition within the gut also occurs among *Caballeronia* strains themselves, driving specificity at a finer taxonomic scale. The stinkbugs *Riptortus pedestris* and *Coreus marginatus*, when reared on the same soil sample, preferentially selected for α- and β-subclade *Caballeronia*, respectively. In a gnotobiotic infection system, representative strains from the α-, β-, and γ-subclades can independently colonize the midgut of both insect species in monoculture. However, in pairwise co-culture infections, each host exhibits selectivity for either α- or β-subclade strains, consistent with patterns observed in the soil inoculation experiment. In *R. pedestris*, we further find that both priority effects and displacement mechanisms shape interspecies competition outcomes. At the molecular level, metabolic capabilities, resistance to antimicrobial peptides, and chemotactic behavior determine symbiont competitive success. In *R. pedestris*, the reproductive fitness benefits conferred by the symbiosis align with the observed strain specificity in the tested strain panel, suggesting a functional link between symbiont selection and host fitness, despite these processes occurring at distinct stages of the symbiotic relationship. Our findings highlight that the gut in Coreoidea species constitutes a multifactorial, species-specific selective environment that contributes to the colonization of the symbiotic midgut region by the best-adapted *Caballeronia* strain.

## Introduction

Since their early evolutionary history, insects have developed heritable interactions with a wide range of microorganisms [1, 2]. These symbionts have expanded the repertoire of capabilities in insects and thereby contributed to their ecological success [3, 4]. To maintain beneficial partnerships, insects have evolved mechanisms that ensure reliable symbiont acquisition and partner fidelity. Most insect symbioses are stably maintained through vertical transmission, passing symbionts from parent to offspring [1, 2]. However, some insects acquire their symbionts from the environment. In such cases, each symbiont-free hatchling must identify and selectively recruit suitable symbionts but avoid harmful or non-cooperative microorganisms.

Partner choice in environmentally transmitted symbioses often involves molecular signaling between host and symbiont, as seen in well-studied systems like rhizobia-legume and *Aliivibrio fischeri*–squid interactions [5]. Insects from the infraorder Pentatomomorpha provide models to study mechanisms governing the establishment of symbiosis by environmental transmission. Many phytophagous Pentatomomorpha have a midgut with a distinctive architecture, which harbors dense populations of a single bacterial species in the crypts of a specialized, posterior gut region, called M4 [6]. Although species from the Pentatomoidea superfamily typically transmit their γ-proteobacterial M4 crypt symbionts vertically, members of the superfamilies Lygaeoidea, Coreoidea, and the family Largidae (within the Pyrrhocoroidea superfamily) harbor environmentally acquired species of the *Burkholderia s.l.* (*sensu lato*) that includes the genera *Caballeronia*, *Paraburkholderia*, and *Burkholderia*. The association of the Pentatomomorpha insects with crypt symbionts increases their fitness by enhancing developmental rate and body size, survival, reproduction, immunity, and capacity for degradation of xenobiotics [7–19].

In the bean bug *Riptortus pedestris*, belonging to the Coreoidea, *Caballeronia* dominates the gut microbiota with >95% prevalence [7]. The gut features a constricted region (CR) between midgut sections M3 and M4 that acts as a selective barrier, permitting only motile and compatible bacteria, such as *Caballeronia insecticola*, to colonize the crypts [20]. This gate closes shortly after initial colonization, preventing late-arriving bacteria from entering [21]. Within the crypts, bacterial competition further filters symbionts, favoring strains with robust metabolic capabilities and resistance to host-derived antimicrobial peptides (AMPs) [22–25].

**Figure 1:**
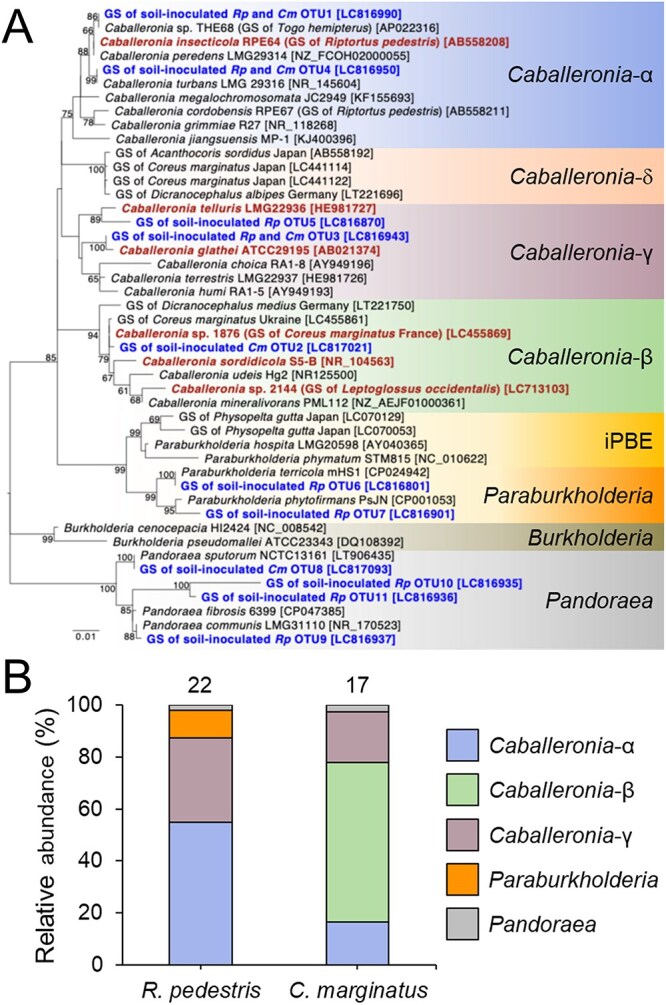
Molecular phylogenetic analysis of gut symbionts of *R. pedestris* and *C. marginatus*. (A) Maximum-likelihood tree of 1285 aligned nucleotide sites of the 16S rRNA gene. Numbers at the tree nodes indicate bootstrap values (%) with 1000 replicates and bootstrap values of more than 50 are shown. Accession numbers in the DNA database (DDBJ/EMBL/GenBank) are shown in square brackets. Gut symbionts of *R. pedestris* (*Rp*) and *C. marginatus* (*Cm*) identified in the soil inoculation test are shown in blue. *Caballeronia* strains used in insect inoculation tests are shown in red. The δ-subclade is also known as “Coreoidea-clade” [15]. GS: Gut symbiont. Scale bar refers to a phylogenetic distance of 0.01 nucleotide substitutions per site. (B) Relative abundance of α-, β-, and γ-*Caballeronia*, *Paraburkholderia*, and *Pandoraea* among gut symbionts of *R. pedestris* and *C. marginatus* in the soil inoculation test*.* The number of investigated insects is shown on top of the graphs, and the corresponding OTUs and their counts are provided in Supplementary Fig. S2 and Supplementary Table S2.

Recent evidence suggests that Coreoidea species may exhibit distinct *Caballeronia* subclade-level specificity, possibly shaped by host-symbiont coevolution or alternatively, by environmental availability of *Caballeronia* subclades in soils of different geographic origin [15]. For instance, *R. pedestris* typically associates with *Caballeronia* of the α-subclade, while the western conifer seed bug *Leptoglossus occidentalis* harbors β- or δ-subclade species across global populations. In the dock bug *Coreus marginatus*, β-subclade associations dominate in Europe, while Japanese populations also carry δ-subclade symbionts (originally placed in the α-subclade, Fig. 1A) [12].

In this study, we experimentally tested whether *R. pedestris* and *C. marginatus* distinguish among *Caballeronia* subclades. First, we analyzed symbiont associations in both insects reared on the same soil. Next, we used gnotobiotic mono- and co-inoculations to assess host preferences for α-, β-, and γ-subclade strains. Finally, we identified key molecular pathways in α-subclade *C. insecticola* that promote its competitive success during gut colonization.

## Materials and methods

### Insect rearing and infection with symbionts

The *R. pedestris* TKS1 inbred line and *C. marginatus* captured in two distinct locations in Gif-sur-Yvette (48°42′17.0″N 2°07′42.2″E) and Bures-sur-Yvette (48°41′52.4″N 2°09′09.3″E), France were reared in the laboratory as described before [12, 26]. Bacteria are listed in Supplementary Table S1 and were cultured as described [24].

For the soil inoculation test, samples of soil from the CNRS campus (48°42′17.0″N 2°07′42.2″E), Gif-sur-Yvette, France were collected in 2019 and sieved with a 2 mm sieve to remove debris. One gram of soil was wetted with 10 ml of water and applied to a cotton pad, which was used as an inoculum for second instar nymphs that are most perceptive to infection by crypt symbionts [27]. Insects were reared until reaching the third instar (5 days postinfection (dpi) for *R. pedestris* and 7 dpi for *C. marginatus*) and their M4 midgut regions were harvested by dissection. DNA was extracted from M4 samples and a clone library analysis of the 16S ribosomal RNA (rRNA) gene was performed as previously described [12]. Multiple alignment of the sequences was performed to assign them to operational taxonomical units (OTU) and to construct a molecular phylogenetic tree as described in the Supplementary Information. The nucleotide sequence data of the 16S rRNA gene obtained in the present study have been deposited in the DDBJ public database with the accession numbers LC816739-LC817101.

In the gnotobiotic infection system, a defined bacterium or set of bacteria is provided to the insects. Inoculation of insects by single fluorescently labeled strains (Supplementary Table S1) or by equally abundant paired strains, each labeled with a different fluorescent protein (Supplementary Table S1), was done as previously by oral administration via cotton pads, wetted with the bacterial suspension [24]. Inoculated second instar nymphs of *R. pedestris* or *C. marginatus* were reared until third instar as above and the symbiont colonization status was assessed in dissected guts by fluorescence microscopy or flow cytometry as described earlier [24]. In pairwise competitions, a competition index (CI) was calculated from the flow cytometry quantification of the competing strains as the ratio of a tested Green Fluorescent Protein (GFP)-strain to a mScarlet-I-strain, normalized by the ratio of the inoculum. Statistical analysis, using a Kruskal–Wallis test, Dunn post hoc test, and Benjamini–Hochberg correction, was performed with RStudio [28].

For fitness measurements in *R. pedestris,* insects were infected as described above with different *Caballeronia* strains and bacteria-free aposymbiotic (apo) insects were obtained by omitting bacteria in the drinking water. Survival rate and developmental time to reach adulthood were reported by daily observation. Morphological parameters were measured on acetone-dehydrated and air-dried 1 day old adults. Egg production in 1 week was counted for individual females each placed together with two males in a container as described before [29]. Statistical analysis, using a Kruskal–Wallis test, Dunn post hoc test, and Benjamini–Hochberg correction, was performed with RStudio [28].

### Phenotypic characterization of *Caballeronia* strains

To measure the velocity of the different strains in liquid Yeast Glucose (YG) medium, time-lapse videos of the bacteria’s movement were recorded using Leica DMI6000 B Inverted Research Microscope (Leica Camera AG, Germany). The tracking and analysis of the motility was done using the TrackMate plugin [30] of Fiji [31].

Combined chemotaxis/motility assays were performed on swimming plates (YG medium with 0.3% agar), inoculated with the tested strains by inserting a suspension of the bacteria into the soft agar layer. Images of the plates were taken at regular time points for around 60 h, and the diameter of the colony growth in the plate was measured on the pictures using Fiji.

For pairwise *in vitro* competition experiments, GFP- and mScarlet-tagged strains (Supplementary Table S1) were mixed together in a 1:1 ratio. Strain mixtures were inoculated either in liquid Minimal Medium (MM) with either glucose or succinate as carbon source, on standard (1.5% agar) YG plates, or on swimming plates as above. Co-cultures were grown for 24 h at 28°C. The cells were harvested in 1X posphate-buffered saline (PBS) buffer from across the growth for liquid cultures and solid agar plates or from the edges only of the growth zone for the swimming plates. The relative number of GFP- and mScarlet-I-labeled bacteria in the harvested cells of each sample was measured by flow cytometry and a CI was calculated as above.

Strain metabolic profiles were analyzed using four 96-well Phenotype Microarray (PM) (Biolog Inc., USA) microplates, which consisted of two microplates of carbon substrates (PM1 and PM2A), one microplate of various nitrogen substrates (PM3B), and one microplate of different phosphorus and sulfur substrates (PM4A). The assays were performed following the manufacturer’s instructions.

Sensitivity assays of the *Caballeronia* strains to AMPs were performed essentially as before and as detailed in the Supplementary Information [25].

### Other methods and detailed procedures

Full descriptions of all above methods as well as the creation of fluorescent protein labeled strains are provided in Supplementary Information.

## Results

### *Coreus marginatus* and *R. pedestris* select *Caballeronia* strains of different subclades from the same soil sample

To determine whether host specificity or geographic preference of *Caballeronia* subclades is involved in the midgut colonization in Coreoidea insects, we performed a soil-inoculation experiment that mimics more closely the natural symbiont acquisition process than the standard laboratory gnotobiotic system of insect colonization with specific, selected bacterial strains. For this, second instar nymphs of *R. pedestris* and *C. marginatus* were reared on the same soil suspension, prepared from a natural prairie soil sample. Bacterial diversity in the midgut crypts of the third instar nymphs was investigated by generating plasmid clone libraries of 16S rRNA gene Polymerase Chain Reaction (PCR) products per insect specimen and sequencing of clones. In a total of 363 sequences from 22 and 17 individuals of *R. pedestris* and *C. marginatus*, respectively, 11 OTUs were obtained, which corresponded to *Caballeronia*, *Paraburkholderia*, and *Pandoraea* spp. (Supplementary Table S2; Supplementary Figs. S1 and S2). The data suggest that the M4 of insect individuals is mostly dominated by a single phylotype (Supplementary Fig. S2).

A molecular phylogenetic analysis based on the 16S rRNA gene, including the sequences of the 11 OTUs, several type strains of *Caballeronia*, *Burkholderia*, *Paraburkholderia*, and *Pandoraea*, as well as previously reported gut symbionts of different Coreoidea insects, placed the *Caballeronia* OTUs in the α-, β-, and γ-subclades (Fig. 1A). No OTU from our sampling was detected within the δ-subclade (also called “Coreoidea-clade” [15]), despite the fact that all known sequences of this subclade are derived from wild specimen of Coreoidea insects, including from Japanese specimen of *C. marginatus* [6, 12, 15, 32]. Although this clade has been detected in the midgut of Coreoidea stinkbugs, it has been rarely detected in other environments. Hence it is considered to be very low abundant in soil. Two OTUs were placed in the *Paraburkholderia* but apart from the previously defined insect-associated, plant-associated beneficial and environmental (iPBE) clade, containing the gut symbionts of *Largidae* species [33–35]. Four other OTUs were in the *Pandoraea* group.

The relative proportion of the OTUs detected in the M4 midguts of *R. pedestris* and *C. marginatus* demonstrates that *Caballeronia* dominated in both insects with respectively 87.5% and 97.5% of the detected crypt strains (Fig. 1B; Supplementary Table S2), in agreement with the known specificity of these insects for *Caballeronia* spp. [7, 12]. However, strains of the α-subclade of *Caballeronia* occupied 56% in *R. pedestris*, but only 16% in *C. marginatus*. In contrast, β-strains showed the opposite tendency, with 61% detected in *C. marginatus*, but none in *R. pedestris*. The γ-strains showed 33% and 20% of occupancy in *R. pedestris* and *C. marginatus*, respectively. Thus, the dominant *Caballeronia* strains were different between *R. pedestris* and *C. marginatus* suggesting a different symbiont specificity at a fine taxonomic scale.

### Colonization of gnotobiotic *R. pedestris* and *C. marginatus* by α-, β-, and γ-subclade *Caballeronia* strains

**Figure 2:**
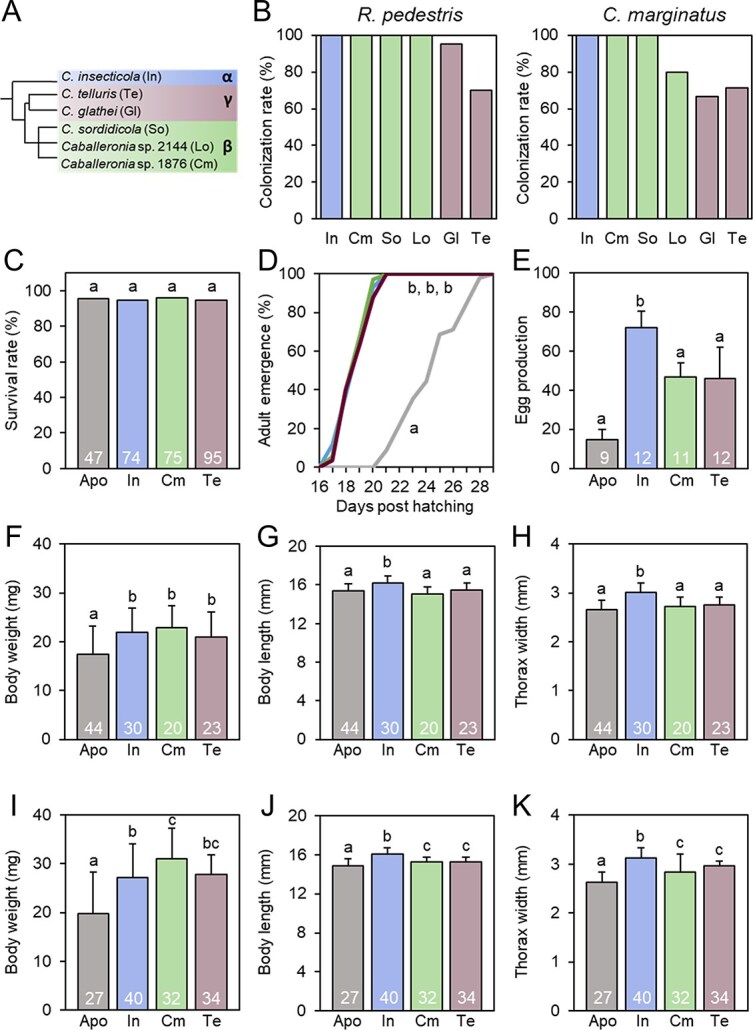
The crypt colonization ability of *R. pedestris* and *C. marginatus* and fitness advantages in *R. pedestris* provided by *Caballeronia* strains. (A) Phylogenetic tree, based on the 16S rRNA gene, of *Caballeronia* strains used in bacterial inoculation tests in *R. pedestris* and *C. marginatus*. Abbreviations of bacterial strain are provided in brackets. (B) Second instar nymphs were inoculated with single strains as indicated. The colonization rate in the midgut crypts of *R. pedestris* and *C. marginatus* is indicated. The third instar nymphs of *R. pedestris* (*n* = 20–59) and *C. marginatus* (*n* ≥ 5) were investigated at 5 dpi and 7 dpi, respectively. The bacterial names are provided in abbreviated form as indicated in A. (C–K) Fitness advantages in *R. pedestris* provided by *Caballeronia* strains. *Riptortus pedestris* individuals were colonized by the indicated *Caballeronia* strains*.* The following fitness parameters were determined: Insect survival rate (C); developmental time until reaching adulthood (D); number of eggs produced during the first week after commencement of egg-laying by one female reared in the presence of two males (E); body weight of males (F); body length of males (G); thorax width of males (H); body weight of females (I); body length of females (J); thorax width of females (K). Standard deviations are indicated by vertical bars. The numbers in the histograms indicate the number of analyzed samples *n*. Different letters indicate significant differences (*P* < .05) determined by a Kruskal–Wallis test with the Dunn post hoc test and *P*-value adjustment with the Benjamini–Hochberg method.

To investigate the ability of *Caballeronia* belonging to the different subclades to colonize the midguts of *R. pedestris* and *C. marginatus*, GFP-labeled strains were independently inoculated to second instar nymphs of *R. pedestris* and *C. marginatus*, and the status of midgut colonization at the third instar nymphs was surveyed by epifluorescence microscopy. The investigated strains included the α-strain *C. insecticola* (isolated from the gut of a *R. pedestris* specimen), three β-strains, Cm1876 (isolated from the gut of a *C. marginatus* specimen), Lo2144 (isolated from the gut of a *L. occidentalis* specimen), and *Caballeronia sordidicola* (type strain DSM17212^T^), and the two γ- strains *Caballeronia telluris* (type strain LMG22936^T^) and *Caballeronia glathei* (type strain JCM10563^T^) (Fig. 2A; Supplementary Table S1) [12, 15]. The gnotobiotic infection test demonstrated that the α- and β-strains displayed an almost 100% colonization ability in the midgut crypts (proportion of tested insects that were infected in the M4 crypts) of both, *R. pedestris* and *C. marginatus*, whereas the colonization rate with the γ-strains was slightly lower than the other two *Caballeronia* subclades, particularly in *C. marginatus* (Fig. 2B). Inspection of the crypt infection by fluorescence microscopy confirmed a regular infection pattern for all tested strains in the two insects except for *C. telluris* (γ-strain) in the *C. marginatus* crypts, which were only partially infected by this strain in some individuals (Supplementary Figs. S2 and S3). Together, these results show that strains of the three subclades have high colonization ability in the midgut of *R. pedestris* and *C. marginatus,* though γ-strains are slightly less efficient.

We determined if selected strains from the α-, β-, and γ-subclades provide fitness advantages to the host insect. This analysis was limited to *R. pedestris* because efficient rearing conditions are available for this insect and fitness advantages derived from the crypt symbionts have been well characterized [7, 29], while the generation of offspring in *C. marginatus* in our rearing conditions was insufficient for multiple experiments. The survival rate, developmental time to reach adulthood, body mass and size of adults, and egg production were determined in *R. pedestris* apo insects or insects infected with *C. insecticola* (α-subclade strain), *Caballeronia* strain Cm1876 (β-subclade strain), or *C. telluris* (γ-subclade strain) (Fig. 2C–K). Insects infected with the three strains showed a nearly 100% survival rate, identical to apo insects, indicating that none of the bacteria had a negative effect on the host (Fig. 2C). The different measured fitness parameters showed that all three tested strains provided an enhanced fitness to the host, compared to apo insects (Fig. 2D–K). However, whereas the developmental time and body mass were similar for the insects infected with the three *Caballeronia* strains, body size and egg production were significantly lower in insects infected with *Caballeronia* strain Cm1876 or *C. telluris* compared to insects carrying the natural crypt symbiont *C. insecticola* (Fig. 2E–K). Thus overall, even if all the tested *Caballeronia* strains can provide fitness services to the host, *C. insecticola* provides superior advantages.

### *Caballeronia* interspecies competition determines the crypt occupancy

Although *Caballeronia* of the three subclades has a good colonization ability in *R. pedestris* and *C. marginatus*, the soil inoculation test demonstrated that respectively α- and β-OTUs were dominant in these two insect species (Fig. 1). To further explore a potential selection at the *Caballeronia* subclade level, pairwise bacteria-bacteria competition assays were conducted. Equal amounts of GFP- and mScarlet-I-labeled strains belonging to different *Caballeronia* subclades (Supplementary Table S1) were inoculated in growth medium or fed to *R. pedestris* and *C. marginatus* nymphs and the relative amount of both strains was determined by flow cytometry after growth in *in vitro* cultures or establishment in the M4 crypts of the insects. In liquid culture, two carbon sources were used, the glycolytic carbon source glucose and the gluconeogenic carbon source succinate. The latter nutrient was used because it was demonstrated that in the crypts, the colonizing bacteria are fed with a gluconeogenic substrate [24, 26]. Besides revealing differences in growth rate between strains, competition in liquid cultures also test for secretion of compounds that are toxic for competing bacteria. Additionally, antagonism between competitors by contact-dependent mechanisms [36] was tested on solid agar plates that favor cell–cell contact.

**Figure 3:**
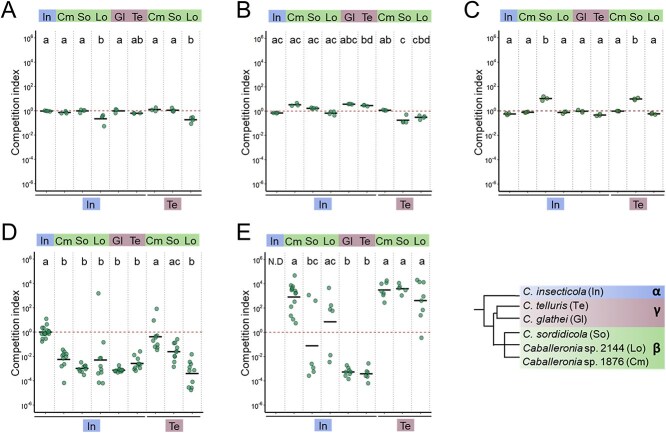
Competition assays of *Caballeronia* strains in culture and in insect midgut crypts. (A and B) CI of *Caballeronia* strains in culture in minimum medium with glucose (A) or succinate (B) as carbon source (*n* = 4). (C) CI of *Caballeronia* strains in growth in contact on solid agar plates with YG medium. (D and E) CI of *Caballeronia* strains in the midgut crypts of *R. pedestris* (*n* = 10) (D), or *C. marginatus* (*n* = 5–14) (E). Midguts of *R. pedestris* and *C. marginatus* were investigated at 5 dpi and 7 dpi, respectively. The used strains are abbreviated according to the key at the bottom right. The CI indicates the ratio of a tested β- or γ-strain to the *C. insecticola* (α) or *C. telluris* (γ) strain, normalized by the ratio of the inoculum (inocula were prepared with close to equal amounts of the two competing strains). Dots and black lines show the CI in individual insects and the mean of the replicates, respectively. Letters indicate significant differences (*P* < .05) using a Kruskal–Wallis test with the Dunn post hoc test and *P*-value adjustment with the Benjamini–Hochberg method.

For *in vitro* control competitions, GFP- and mScarlet-I-labeled *C. insecticola* clones, both derivatives of the *Riptortus pedestris* endosymbiont (RPE) RPE75 strain (Supplementary Table S1), kept each other in close balance in the two liquid media and the solid medium (Fig. 3A–C), indicating that the introduction of fluorescent markers, carried on a Tn*7* transposon inserted in the genome, has no noticeable effect on the fitness of the bacteria. In all tested strain combinations in the three *in vitro* set-ups, strain-pairs mostly kept each other in balance with only minor deviations from equal abundance after 24 h of growth (|CI| ≤ 10^1^) (Fig. 3A–C). Thus, the pairwise competition assays in culture revealed no strong growth differences or antagonisms between *Caballeronia* strains.

Contrary to the cultures, pairwise combinations in the midgut crypts of the two tested insects showed mostly strong competitive interactions whereby one strain outcompeted the other (|CI| > 10^2^). In the case of *R. pedestris*, the β-subclade strains were outcompeted by the α-strains in the midgut crypts in all tested cases (Fig. 3D). The same pairwise competitions provided in *C. marginatus* very different outcomes. Here, the β-strain Cm1876 outcompeted the α-strain *C. insecticola*, while the two other combinations resulted in a stochastic crypt colonization outcome wherein each insect was infected almost exclusively by either one of the paired strains in a random fashion (Fig. 3E). Also in the case of β- versus γ-competitions, the outcomes were strongly contrasted in the two insect species (Fig. 3D and E). The γ-strain was significantly outcompeted by the β-strains in *C. marginatus*. In contrast, the γ-strain outcompeted two of the β-strains in *R. pedestris* while the other γ–β confrontation between *C. telluris* and strain Cm1876 was the only observed case where the two strains could coexist in the crypts of the same insects. In both insect species, the γ-strains were outcompeted by the α-strain (Fig. 3D and E). Several of the competitions in *C. marginatus* showed a higher variability in the CI between insect individuals than in *R. pedestris*, suggesting a non-negligible influence of stochastic picking up of symbionts in the former species.

Collectively, the above results demonstrate that the two stinkbug species have different selectivity for the *Caballeronia* subclade strains that they host in the M4 midgut crypts. Overall, the specificity can be summarized as α > γ > β for *R. pedestris* and β > α > γ for *C. marginatus* (Fig. 3), which is in very good correspondence to the specificity suggested by the soil inoculation experiment (Fig. 1).

### Dynamics of interspecies competitions reveal different underpinning mechanisms

**Figure 4:**
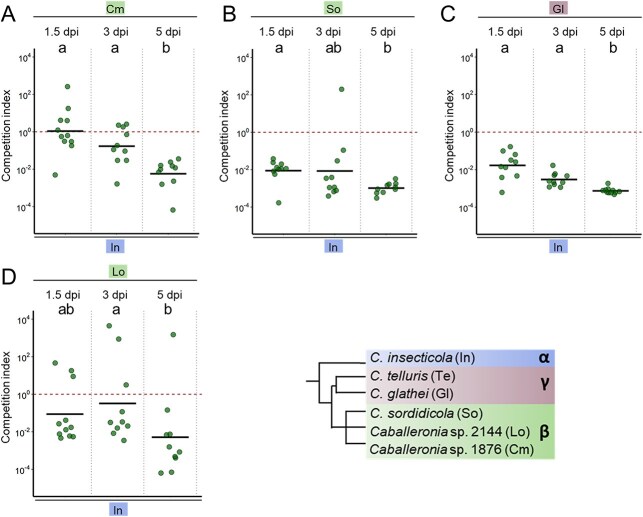
Dynamics of competition of *Caballeronia* strains in midgut crypts of *Riptortus pedestris*. (A) CI of Cm versus In. (B) CI of So versus In. (C) CI of Gl versus In. (D) CI of Lo versus In. Competitions were determined in the midgut crypts of *R. pedestris* (*n* = 10) at 1.5, 3 and 5 dpi. The CI indicates the ratio of the two tested strains, normalized by one of the inoculum. Dots and black lines show the CI in individual insects and the mean of the replicates, respectively. The used strains are abbreviated according to the key at the bottom right. Different letters indicate statistically significant differences (*P* < .05). Statistical significance was analyzed by Kruskal–Wallis test, Dunn post hoc test and *P*-value adjustment with the Benjamini–Hochberg method.

Out-competition of one strain by another in co-infection could be the result of priority effects whereby the timing of arrival in the crypt area will determine which strain will win the competition or alternatively, competing strains might initially coexist in the crypts followed by displacement of one of the strains. In previous confrontation experiments between the α-strain *C. insecticola* and more distantly related *Paraburkholderia* or *Pandoraea* spp., it was shown that initially strains coexisted in the crypts and that *C. insecticola* subsequently displaced the competitor [22]. We analyzed four of the above interactions if we could detect evidence for priority effects or displacement mechanisms. The dynamics of competition during M4 crypt colonization in *R. pedestris* was followed over time after co-infections of the insects with *C. insecticola* (α-strain) together with either strain Cm1876 (β-strain), *C. sordidicola* (β-strain), strain Lo2144 (β-strain), or *C. glathei* (γ-strain) (Fig. 4). The M4 community was analyzed at an early time point (1.5 dpi), an intermediate time point (3 dpi), and a late time point (5 dpi). Overall, three patterns of colonization dynamics could be distinguished. In the case of the *C. insecticola*/Cm1876, initially the two strains coexist in the midgut crypts and gradually, *C. insecticola* displaces the Cm1876 strain (Fig. 4A). In contrast, in the case of the *C. insecticola*/*C. sordidicola* and *C. insecticola*/*C. glathei* interactions, *C. insecticola* dominates from the early stages, a pattern that suggests a priority effect (Fig. 4B and C). Finally, the colonization pattern of the *C. insecticola*/Lo2144 competition suggests a mixed mechanism whereby a priority effect leads to a majority of either one of the strains in the crypts followed by a displacement of the Lo2144 strain by *C. insecticola* (Fig. 4D).

### Bacterial factors contributing to interspecies competition

**Figure 5:**
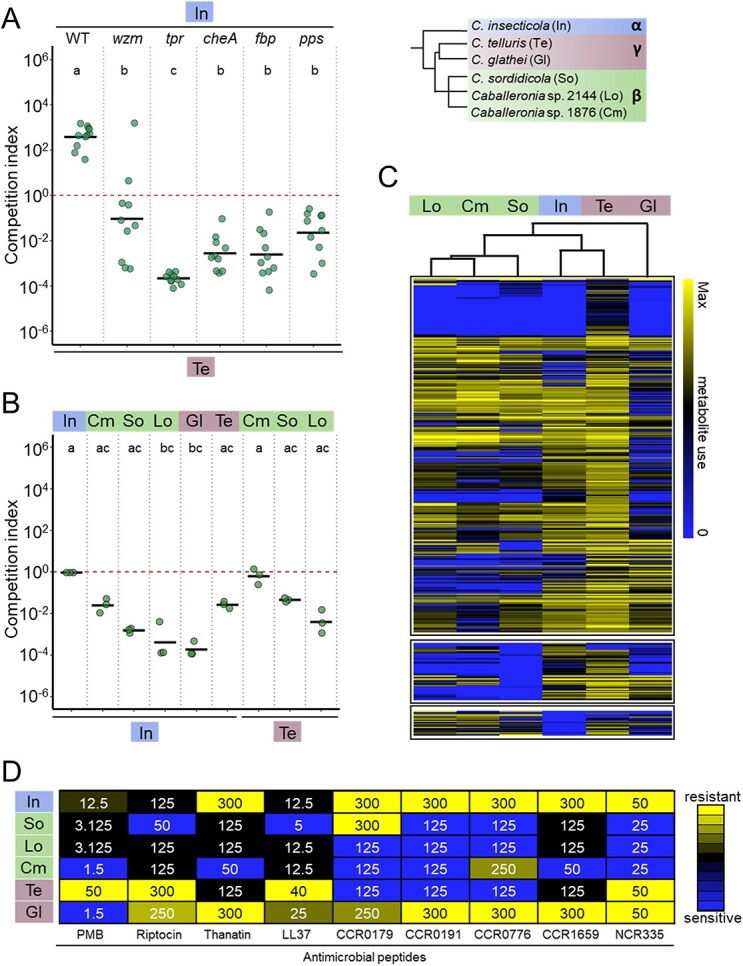
Bacterial functions contributing to interspecies competition. (A) Competition assay of *C. telluris* (γ) and *C. insecticola* (α) wild type (WT) and mutants in midgut crypts of *R. pedestris*. An equal mixture of mScarlet-I-labeled *C. telluris* (γ) and GFP-labeled *C. insecticola* mutants was fed to *R. pedestris* and the relative abundance of mScarlet-I- and GFP-labeled bacteria colonizing the midgut crypts at 5 dpi (*n* = 10) was determined. The CI indicates the ratio of the *C. telluris* strain to the tested *C. insecticola* WT or mutant strains, normalized by the ratio of the inoculum. The *tpr* and *wzm* mutants have reduced AMP resistance. The *cheA* mutant has lost chemotaxis and the *fbp* and *pps* mutants cannot perform gluconeogenesis. Dots and black lines show the CI in individual insects and the mean of the replicates, respectively. Different letters indicate statistically significant differences (*P* < .05). Statistical significance was analyzed by Kruskal–Wallis test, Dunn post hoc test, and *P*-value adjustment with the Benjamini–Hochberg method. (B) Combined motility and chemotaxis competition assays on 0.3% YG agar swimming plates. GFP and mScarlet-I-labeled strains were inoculated in a 1:1 ratio in the center of the plates and cells were harvested after 24 h growth at the edge of the growth zone for measurement of the relative abundance of the GFP and mScarlet-I-labeled strains. (C) Heatmaps and hierarchical cluster analysis of metabolite usage by *Caballeronia* strains using Biolog Phenotype MicroArrays. Metabolites are in rows and strains in columns. The top cluster shows the metabolic capacity of strains for all metabolites used by *C. telluris*. The middle cluster contains metabolites used by *C. insecticola* but not by one or more β-*Caballeronia* strains. The lower cluster contains the metabolites used by the β-*Caballeronia* strains but not by *C. insecticola*. The full dataset of 384 conditions is shown in Supplementary Fig. S6. The compound order in the three clusters is available in the Supplementary Data Set 1. (D) Resistance of *Caballeronia* strains to AMPs. The heat map shows the minimal concentrations of growth inhibition of the indicated wild-type and mutant strains by the listed AMPs. Minimal concentrations are indicated in μg/ml. The color key of the heat maps in panels C and D are indicated at their right. The used strains in all panels are abbreviated according to the key at the top right.

Several molecular mechanisms underlying the efficient *R. pedestris* M4 midgut colonization capacity of *C. insecticola* have been revealed. Among them are the use of host-derived carbon sources via gluconeogenesis, chemotaxis, and resistance to AMPs. Mutants of *C. insecticola* in the genes *fbp* and *pps* (gluconeogenesis) [24], *cheA* (chemotaxis; Supplementary Fig. S4) [37], or *wzm* and *tpr* (AMP resistance) [25] are still capable to colonize the *R. pedestris* M4 crypts but less efficiently than the parental wild-type strain and these mutants are fully outcompeted by the wild type. To determine whether factors controlled by these five genes are also involved in interspecific bacteria-bacteria competition in the midgut of *R. pedestris,* we conducted competition assays in *R. pedestris* confronting the five mutants with the γ-strain *C. telluris*. As above, the wild-type *C. insecticola* strongly outcompeted *C. telluris* while all five mutants were severely affected to various degrees (Fig. 5A). This result indicates that the superior performance of *C. insecticola*, compared to *C. telluris*, for *R. pedestris* midgut colonization is multifactorial, depending at least on nutritional, stress-response, and chemotactic adaptations. To further elaborate this hypothesis, we compared these three parameters directly in the members of our *Caballeronia* test panel.

The swimming velocity of the six strains was determined in exponential phase bacteria growing in rich medium using videos taken by fluorescence microscopy. All strains were motile showing moderate variations in the mean velocity (Supplementary Fig. S5A). Next, the chemotactic performance of the strains was compared using swimming plates in which bacteria are inoculated in soft agar and move away from the inoculation point through their combined motility and chemotactic activity. The measurement of the diameter of growing colonies in function of time highlighted differences between strains, suggesting variable chemotactic abilities (Supplementary Fig. S5B). Pairwise competitions on swimming plates confirmed that the more efficient chemotactic strains outcompeted the less efficient ones at the front of the growing community (Fig. 5B). The CIs on the swimming plates correlated well with the corresponding CIs in the gut of *R. pedestris* (Fig. 3D), suggesting that chemotaxis is a strong contributor to the specificity in this species. In contrast, the pattern of CIs in the swimming plates (Fig. 5B) is different from the CIs in the *C. marginatus* gut (Fig. 3E), suggesting that in this insect, mechanisms, other than motility and chemotaxis, are more important in symbiont selection.

The metabolic capacities of the strains in the test panel were profiled with Biolog Phenotype MicroArrays [38]. Testing the use of hundreds of potential carbon, nitrogen, phosphorous, and sulfur sources revealed that the six strains have distinguishable metabolite usage patterns whereby the three tested β-*Caballeronia* strains are most similar to each other while the α- and two γ-subclade strains are each different (Supplementary Fig. S6). The γ-subclade strain *C. telluris* seems to metabolize the largest set of compounds and only few compounds that are metabolized by the other strains are not processed by it (Fig. 5C, top cluster). Nevertheless, this strain is not dominating the α-subclade strain in *R. pedestris* or the β-*Caballeronia* strains in *C. marginatus* (Fig. 3D and E), indicating that the superior metabolic capacity of *C. telluris* is not sufficient for dominating in the crypts of the two stinkbug species. In contrast, the α-strain *C. insecticola* metabolizes several compounds that are not processed by the β-*Caballeronia* strains (Fig. 5C, middle cluster), which could contribute to the superior colonization capacity of *C. insecticola* in *R. pedestris* relative to the β-strains. At the opposite, the β-strains have also metabolic capacities that are absent in *C. insecticola* (Fig. 5C, bottom cluster) but as these capacities are mostly shared by the tested β-strains, while they do not have the same competitive advantage to *C. insecticola* in the colonization of the *C. marginatus* gut (Fig. 3E), it is not straightforward to correlate these metabolic capacities with gut colonization. Taken together, the *Caballeronia* strains have distinct metabolic activities, which could contribute to the efficiency of crypt colonization although, in the absence of knowledge on the specific identify of crypt metabolites, they are not easily correlated with the observed competitive behavior in the *R. pedestris* and *C. marginatus* crypts.

We tested the resistance of the *Caballeronia* strains of the test panel against a set of AMPs, including crypt-specific cysteine rich peptide (CCR) AMPs produced in the posterior midgut of *R. pedestris* [25], together with the innate immunity-related AMPs thanatin and riptocin [19], mammalian LL37 [39], plant NCR335 [40], and bacterial polymyxin B. The three β-strains as well as the γ-clade strain *C. telluris* were relatively more sensitive to the tested peptides, and in particular to the CCR peptides, than the α-strain *C. insecticola* or the γ-strain *C. glathei* (Fig. 5D). Thus, AMP resistance can contribute to the dominance of *C. insecticola* in the gut crypts relative to the β-strains or the γ-strain *C. telluris*. In contrast, the dominance of *C. insecticola* over *C. glathei* is probably independent of AMP resistance because both strains have similar resistance patterns. Moreover, the β-strain Cm1876 outcompetes *C. insecticola* in the crypts of *C. marginatus*, despite its lower resistance to AMPs. Thus, the dominance of the β-*Caballeronia* strain Cm1876 in the *C. marginatus* crypts seems to be independent of its overall metabolic performance, motility and chemotaxis, and AMP resistance, which suggests that this bacterium has a yet to be identified adaptation for the crypt colonization in this particular host.

## Discussion

Field-collected *R. pedestris* and *C. marginatus* are both predominantly colonized by *Caballeronia* spp. in their M4 midgut region [6, 7, 12, 41], a pattern confirmed in our study in insects of these two species reared on the same French soil sample. Our result also matches earlier observations in *R. pedestris* with soil samples from South Korea and Japan [42]. However, even if the two insect species displayed the same specificity at the genus level, we discovered a marked difference between them at a finer taxonomic level. Whereas α-*Caballeronia* dominated in *R. pedestris* infected with soil bacteria, β-*Caballeronia* prevailed in *C. marginatus*. Gnotobiotic infection experiments corroborated these species-dependent specificities for *Caballeronia* subclades. Mono-inoculation experiments in the gnotobiotic system showed strains of all *Caballeronia* subclades could colonize either host. However, co-inoculation revealed competitive hierarchies between subclade strains: the α-*Caballeronia* is more competitive than the tested β- and γ-strains, and the γ-strains more than β-strains to colonize the symbiotic organ of *R. pedestris*; in contrast, β-strains have an advantage for the colonization of *C. marginatus* over α- and γ-strains, and α- over γ-strains. These findings confirm that species-specific subclade specificity, observed in natural specimens [12–15], are at least driven by host-bacteria compatibility and partner-choice mechanisms. We observed that less competitive strains still colonized some individuals reared on soil, likely due to ecological drift when environmental symbiont abundance is low and insects can pick up stochastically less-preferred symbiont strains [43]. Possibly, also in the gnotobiotic infection system in *C. marginatus*, stochastic symbiont selection is happening as suggested by the observed variability in the competition experiments between several of the tested strain pairs. The importance of ecological drift suggests that besides partner choice mechanisms, geographic distribution of *Caballeronia* subclades can also contribute to observed colonization patterns in wild insects.

Subclade-level specificity is also seen in other Coreoidea bugs. For example, *Leptoglossus occidentalis* typically hosts β- or δ-*Caballeronia*, even when co-infected with α-strains [15]. However, specificity varies within the Pentatomomorpha. Largidae species, for example, are selectively colonized by *Paraburkholderia* of the iPBE subclade rather than *Caballeronia* [33–35], whereas Blissidae and Bertidae, are more permissive, accepting species of multiple genera of the *Burkholderia s.l.* [6, 13, 44–47].

Symbiont specificity within the Pentatomomorpha echoes other environmentally transmitted symbioses, like rhizobia-legume or squid-*Aliivibrio* associations, where host-symbiont compatibility is mediated by molecular signaling and variations on the conserved theme of signals account for symbiont specificity [48, 49]. Likewise, we can expect that signal production and recognition are fundamental in the selection of the midgut crypt symbionts by the stinkbugs. Though the specific chemical cues in stinkbug-*Caballeronia* interactions remain unidentified and their identification is a crucial future challenge, our findings suggest that tested *Caballeronia* strains produce conserved signals while subtle differences in signal production and receptor recognition between subclades may guide symbiont selection. The here inferred chemotactic cues produced in the gut and their recognition by chemotaxis receptors of the gut bacteria may constitute one example of a signaling system controlling the stinkbug-*Caballeronia* symbiosis.

Beyond signaling, we show here that microbial competition in the gut strongly shapes the strain composition in the crypts. Crypt colonization begins with a bottleneck at the CR, limiting passage to a few thousand bacteria into the crypt region [20, 21]. Rapid expansion of this founding population to ~10^7^ cells follows, offering two key windows for competition to operate: early priority effects during CR passage and establishment of the founding population and later displacement during outgrowth of the founding population.

Microorganisms generally compete in two ways: indirectly, through adaptation to the environment, or directly, by harming each other via chemical warfare [50]. Direct competition involves secreting antimicrobials like bacteriocins or injecting toxins using systems, such as Type VI secretion (T6SS) or contact-dependent inhibition [36]. Although culture experiments showed no clear antagonism between tested strains, antimicrobial, or toxin-mediated competition still may occur in the midgut crypts. For instance, *C. insecticola* possesses a T6SS that could target competing strains in the crypts, though evidence is still lacking: T6SS genes were downregulated in single-strain infections [26], and T6SS mutants showed no competitive disadvantage against *Pandoraea* or *Paraburkholderia* strains [22].

In contrast to direct competition, indirect competition likely plays a dominant role in midgut crypt colonization and here we identified three mechanisms. One of them is chemotaxis, guiding bacteria toward the M4 midgut region. Flagellar motility is essential for entry into the crypt region in *R. pedestris* through the CR [20, 51, 52]. We found that a *cheA* mutant of *C. insecticola*, though able to colonize alone, is outcompeted by its wild type and by *C. telluris*—which itself is usually outcompeted by the wild-type *C. insecticola*. This suggests that chemotactic efficiency influences competitive success. Supporting this, *Caballeronia* strains of the test panel showed significant differences in motility and chemotaxis, which correlated with competition outcomes *in vitro* that resembled strongly competition outcomes in the crypts of *R. pedestris* (Fig. 5B vs. Fig. 3D). Priority effects observed during infection may result from faster strains reaching the crypts earlier and establishing dominance, especially as the CR closes after the passage of the initial colonizers [21], preventing late arrivers from entering.

Indirect competition may also depend on the ability of strains to adapt to the crypt environment, affecting their growth within the crypt community. Because crypt bacteria feed on metabolites secreted by the crypt epithelium rather than ingested food, due to the crypt’s disconnection from the anterior digestive midgut region [20, 26], nutrient availability in crypts is probably limited in both quantity and diversity. Thus, bacterial growth competition, particularly in the case of displacement of one strain by another, likely hinges on their metabolic gene repertoire for harvesting crypt nutrients. Metabolic phenotyping of the tested *Caballeronia* strains indeed revealed their distinct capabilities. For example, we previously showed that gluconeogenesis and taurine and inositol harvesting contribute to *C. insecticola’*s gut fitness, with mutants in these pathways able to colonize but outcompeted by wild-type strains [24]. Here, we demonstrate that gluconeogenesis mutants also lose the ability to outcompete an otherwise less efficient *Caballeronia* strain, supporting the idea that available nutrient resources in the crypts and matching metabolic capacities in the crypt colonizers determine the outcome of competitions. Differences in crypt nutrients between *R. pedestris* and *C. marginatus*, which feed on soybean seeds [53] and *Rumex* seeds [54] respectively, likely contribute to their specificity for different *Caballeronia* subclades, as their distinct diets are expected to influence crypt metabolite composition.

Stress conditions in the crypt environment can limit bacterial growth and thus that potentially regulates interspecies competitions. The *R. pedestris* crypts produce massively a high variety of AMPs, known as CCRs that can kill or inhibit growth of bacteria [25]. Typical crypt colonizing bacteria are resistant to these peptides but CCR-sensitive mutants of *C. insecticola*, such as the here tested *wzm* and *tpr* mutants, have a strongly reduced fitness for crypt colonization and are outcompeted by wild-type *C. insecticola* or by a competing species [25; this study]. The tested *Caballeronia* strains have distinct resistance profiles to CCRs. Overall, this suggest that the adaptation of candidate gut symbionts to grow in the presence of the CCRs contributes to the selection process during competitive crypt colonization. Moreover, *R. pedestris* and *C. marginatus* possibly produce different panels of CCRs in order to select their matching microbial partners.

The majority of our tested competitions results in a “winner takes all” outcome, with one of the competitors dominating and the other one nearly eliminated (|CI| > 10^2^). This observation reflects well the colonization pattern seen in insects reared on soil or in wild-captured insects of different bug species, which have midgut crypts that are mostly dominated by a single OTU [this work; 12, 13, 33, 29, 42, 45, 55]. Because the bacterial population in the crypts is generated by a small founding population that multiplies within the crypts [21], the nearly mono-strain composition of the final crypt population needs to be explained by either strong differences in the number of bacteria of competing strains composing the crypt founding community, despite an equal composition in the inoculum in our experimental competitions (e.g. due to different efficiency of strains in passage through the CR) or by strong differences in the growth rate of the strains within the crypts (e.g. due to differences in the adaptations to the nutritional or stress conditions in the crypts).

Although all tested strains provide an enhanced fitness to the host compared to apo insects, the most competitive symbiont of *R. pedestris* provides higher reproductive benefits to the insect than the less favored strains, despite the fact that the selection of the symbiont is happening in the early life of the host, well before the host can assess the benefits received from the symbiont during adulthood and reproduction. Other studies on related bug-*Caballeronia* associations reported a nearly the same degree of host benefit provided by more or less distantly related symbionts [8, 22, 56]. However, these studies measured fitness parameters like survival, developmental time, and body size, while the strongest difference that we have detected between strains is egg production, which was not compared between symbiont strains in the other studies. Possibly, determining the reproduction fitness in other strain comparisons, or other parameters like immune priming [19], could reveal differences in strain performances also in other cases. Admittedly, our observation of larger fitness provided by the most competitive strain is based on comparing a small number of strains comprising one competitive and two less-competitive ones. The hypothesis of a connection between symbiont selection and fitness will require further testing involving a larger panel of strains.

The possible selection of optimal symbionts by *R. pedestris* contrasts starkly with the selection of rhizobium symbionts by legumes, which are unable to pick out from the soil efficient nitrogen fixing symbionts from inefficient strains because the nitrogen fixation per se is not active during the selection and infection process but only at the late stage of the interaction when the nodule is formed and occupied with the selected rhizobium strain [57, 58]. However, at the level of the whole plant that carries many nodules, the host applies sanctions to the least efficient nodules. Because sanctioned nodules contain fewer viable bacteria than non-sanctioned ones, efficient nitrogen-fixing symbionts nevertheless progressively outcompete less efficient strains [59, 60]. The proposed alignment of the symbiont-host specificity with the host benefits obtained from the symbiont in *R. pedestris* suggests a different scenario. Contrary to the rhizobia-legume symbiosis, the insects can potentially monitor already in the infecting bacteria the capacity to produce the services that will contribute ultimately to the host fitness at a later stage. These services are at present not clearly defined at the molecular level and their identification will be helpful to better understand the details of symbiont selection in these insects. In addition, the crypt symbionts could not only deliver specific metabolites to the host but also need to provide bacterial biomass to replace crypt bacteria that are consumed in the M4B region, an anterior subregion of M4 whose function is to digest symbiont cells and absorb the derived nutrients that support insect development [26]. In that case, a *Caballeronia* strain with higher proliferation efficiency in the crypts could dominate the bacteria–bacteria competition within the crypts in the early stage of the interaction and supply in a late stage substantial more bacterial biomass, resulting in more nutritional benefits to the host.

## Supplementary Material

Lextrait_et_al_2025_Supplementary_Information_revision_3_wraf240

## Data Availability

The nucleotide sequence data of the 16S rRNA gene obtained in the present study have been deposited in the DDBJ public database with the accession numbers LC816739-LC817101. All other data generated or analyzed during this study are included in this published article and its supplementary information files.
