# Supplementary material for: Strict gut symbiont specificity in Coreoidea insects governed by interspecies competition within Caballeronia strains
Source: ISME J. 2025 Nov 13;19(1):wraf240. doi: 10.1093/ismejo/wraf240 (PMC12632191; doi:10.1093/ismejo/wraf240)
Supplement: Lextrait_et_al_2025_Supplementary_Information_revision_3_wraf240 [file lextrait_et_al_2025_supplementary_information_revision_3_wraf240.pdf]

# SUPPLEMENTARY INFORMATION

## Strict gut symbiont specificity in Coreoidea insects governed by interspecies competition within *Caballeronia*

Gaelle Lextrait[1], Srotoswini Joardar[1,2], Raynald Cossard[1], Yoshitomo Kikuchi[3], Tsubasa Ohbayashi[1,4], Peter Mergaert[1]

[1]Université Paris-Saclay, CEA, CNRS, Institute for Integrative Biology of the Cell (I2BC), 91198 Gif-sur-Yvette, France
[2]Department of Biology, Ludwig-Maximilians-Universität München (LMU), 82152 Munich, Germany
[3]Bioproduction Research Institute, National Institute of Advanced Industrial Science and Technology (AIST), Hokkaido Center, 062-8517 Sapporo, Japan
[4]Institute for Agro-Environmental Sciences, National Agriculture and Food Research Organization (NARO), 305-8604 Tsukuba, Japan

Correspondence to PM peter.mergaert@i2bc.paris-saclay.fr or TO ohbayashi.tsubasa023@naro.go.jp

## Content

Supplementary Figures Page 2

Supplementary Tables Page 11

Supplementary Materials and Methods Page 13

# SUPPLEMENTARY FIGURES

```
OTU6 [LC816801]  Paraburkholderia  ATTGAACGCTGGCGGCATGCCTTACACATGCAAGTCGAACGGCAGCACGGGGGC--AACC
OTU7 [LC816901]  Paraburkholderia  ATTGAACGCTGGCGGCATGCCTTACACATGCCAGTCGAACGGCAGCACGGGGGC--AACC
OTU1 [LC816990]  Caballeronia-α    ATTGAACGCTGGCGGCATGCCTTACACATGCAAGTCGAACGGCAGCACGGGGGC--AACC
OTU4 [LC816950]  Caballeronia-α    ATTGAACGCTGGCGGCATGCCTTACACATGCAAGTCGAACGGCAGCACGGGGGC--AACC
OTU2 [LC817021]  Caballeronia-β    ATTGAACGCTGGCGGCATGCCTTACACATGCAAGTCGAACGGCAGCACGGGGGC--AACC
OTU3 [LC816943]  Caballeronia-γ    ATTGAACGCTGGCGGCATGCCTTACACATGCAAGTCGAACGGCAGCACGGGGGC--AACC
OTU5 [LC816870]  Caballeronia-γ    ATTGAACGCTGGCGGCATGCCTTACACATGCAAGTCGAACGGCAGCACGGGGGC--AACC
OTU8  [LC817093] Pandoraea         ATTGAACGCTGGCGGCATGCCTTACACATGCAAGTCGAACGGCAGCACGGGTGCTTGCAC
OTU9 [LC816937]  Pandoraea         ATTGAACGCTGGCGGCATGCCTTACACATGCAAGTCGAACGGCAGCACGGGTGCTTGCAC
OTU10 [LC816935] Pandoraea         ATTGAACGCTGGCGGCATGCCTTACACATGCAAGTCGAACGGCAGCACGGGTGCTTGCAC
OTU11 [LC816936] Pandoraea         ATTGAACGCTGGCGGCATGCCTTACACATGCAAGTCGAACGGCAGCACGGGTGCTTGCAC
                                   ******************************* ******************* **     *

OTU6 [LC816801]  Paraburkholderia  CTGGTGGCGAGTGGCGAACGGGTGAGTAATACATCGGAACGTGTCCTGTAGTGGGGGATA
OTU7 [LC816901]  Paraburkholderia  CTGGTGGCGAGTGGCGAACGGGTGAGTAATACATCGGAACGTGTCCTGTAGTGGGGGATA
OTU1 [LC816990]  Caballeronia-α    CTGGTGGCGAGTGGCGAACGGGTGAGTAATACATCGGAACGTGTCCTGTAGTGGGGGATA
OTU4 [LC816950]  Caballeronia-α    CTGGTGGCGAGTGGCGAACGGGTGAGTAATACATCGGAACGTGTCCTGTAGTGGGGGATA
OTU2 [LC817021]  Caballeronia-β    CTGGTGGCGAGTGGCGAACGGGTGAGTAATACATCGGAACGTGTCCTGCAGTGGGGGATA
OTU3 [LC816943]  Caballeronia-γ    CTGGTGGCGAGTGGCGAACGGGTGAGTAATACATCGGAACGTGTCCTGTAGTGGGGGATA
OTU5 [LC816870]  Caballeronia-γ    CTGGTGGCGAGTGGCGAACGGGTGAGTAATACATCGGAACGTGTCCTGGAGTGGGGGATA
OTU8  [LC817093] Pandoraea         CTGGTGGCGAGTGGCGAACGGGTGAGTAATATATCGGAACGTACCTTGTAGTGGGGGATA
OTU9 [LC816937]  Pandoraea         CTGGTGGCGAGTGGCGAACGGGTGAGTAATACATCGGAATGTACCTTGTAGTGGGGGATA
OTU10 [LC816935] Pandoraea         CTGGTGGCGAGTGGCGAACGGGTGAGTAATACATCGGAATGTACCTTGTAGTGGGGGATA
OTU11 [LC816936] Pandoraea         CTGGTGGCGAGTGGCGAACGGGTGAGTAATACATCGGAATGTACCTTGTAGTGGGGGATA
                                   ******************************* ******* **  * ** ***********

OTU6 [LC816801]  Paraburkholderia  GCCCGGCGAAAGCCGGATTAATACCGCATACGCTCTGCGGAGGAAAGCGGGGGCTCCTTC
OTU7 [LC816901]  Paraburkholderia  GCCCGGCGAAAGCCGGATTAATACCGCATACGATCTGTGGATGAAAGCGGGGGATCTTC-
OTU1 [LC816990]  Caballeronia-α    GCCCGGCGAAAGCCGGATTAATACCGCATACGATCTACGGAAGAAAGCGGGGGATCCTTC
OTU4 [LC816950]  Caballeronia-α    GCCCGGCGAAAGCCGGATTAATACCGCATACGATCTACGGAAGAAAGCGGGGGATCTTC-
OTU2 [LC817021]  Caballeronia-β    GCCCGGCGAAAGCCGGATTAATACCGCATACGATCTACGGAAGAAAGCGGGGGATCCTTC
OTU3 [LC816943]  Caballeronia-γ    GCCCGGCGAAAGCCGGATTAATACCGCATACGATCTACGGAAGAAAGCGGGGGATCTTC-
OTU5 [LC816870]  Caballeronia-γ    GCCCGGCGAAAGCCGGATTAATACCGCATACGCTCTACGGAGGAAAGGGGGGGATCGCA-
OTU8  [LC817093] Pandoraea         GCTCGGCGAAAGCCGGATTAATACCGCATACGCTCTGAGGAGGAAAGCGGGGGACCTTC-
OTU9 [LC816937]  Pandoraea         GCTCGGCGAAAGCCGGATTAATACCGCATACGCTCTGAGGAGGAAAGCGGGGGACCTTC-
OTU10 [LC816935] Pandoraea         GCTCGGCGAAAGCCGGATTAATACCGCATACGCTCTGAGGAGGAAAGCGGGGGACCTTC-
OTU11 [LC816936] Pandoraea         GCTCGGCGAAAGCCGGATTAATACCGCATACGCTCTGAGGAGGAAAGCGGGGGACCTTC-
                                   ** ***************************** ***  *** ***** *****  *    

OTU6 [LC816801]  Paraburkholderia  GGGACCTCGCGCTACAGGGGCGGCCGATGGCAGATTAGCTAGTTGGTGGGGTAAAGGCCT
OTU7 [LC816901]  Paraburkholderia  -GGACCTCGCGCTACAGGGGCGGCCGATGGCAGATTAGCTAGTTGGTGGGGTAAAGGCCT
OTU1 [LC816990]  Caballeronia-α    GGGACCTCGCGCTATAGGGGCGGCCGATGGCAGATTAGCTAGTTGGTGGGGTAAAGGCCT
OTU4 [LC816950]  Caballeronia-α    -GGACCTCGCGCTGCAGGGGCGGCCGATGGCAGATTAGCTAGTTGGTGGGGTAAAGGCCT
OTU2 [LC817021]  Caballeronia-β    GGGACCTCGCGCTATAGGGGCGGCCGATGGCAGATTAGCTAGTTGGTGGGGTAAAGGCCT
OTU3 [LC816943]  Caballeronia-γ    -GGACCTCGCGCTATAGGGGCGGCCGATGGCAGATTAGCTAGTTGGTGGGGTAAAGGCCT
OTU5 [LC816870]  Caballeronia-γ    -AGACCTCTCGCTCAAGGGGCGGCCGATGGCAGATTAGCTAGTTGGTGGGGTAAAGGCCT
OTU8  [LC817093] Pandoraea         -GGGCCTCGCGCTACAAGAGCGGCCGATATCAGATTAGCTAGTTGGTGGGGTAAAAGCTT
OTU9 [LC816937]  Pandoraea         -GGGCCTCGCGCTACAAGAGCAGCCGATGTCAGATTAGCTAGTTGGTGGGGTAAAAGCTC
OTU10 [LC816935] Pandoraea         -GGGCCTCGCGCTACAAGAGCAGCCGATGTCAGATTAGCTAGTTGGTGGGGTAAAAGCTC
OTU11 [LC816936] Pandoraea         -GGGCCTCGCGCTACAAGAGCAGCCGATGTCAGATTAGCTAGTTGGTGGGGTAAAAGCTC
                                     * **** ****  * * ** ******  ************************* **  

OTU6 [LC816801]  Paraburkholderia  ACCAAGGCGACGATCTGTAGCTGGTCTGAGAGGACGACCAGCCACACTGGGACTGAGACA
OTU7 [LC816901]  Paraburkholderia  ACCAAGGCGACGATCTGTAGCTGGTCTGAGAGGACGACCAGCCACACTGGGACTGAGACA
OTU1 [LC816990]  Caballeronia-α    ACCAAGGCGACGATCTGTAGCTGGTCTGAGAGGACGACCAGCCACACTGGGACTGAGACA
OTU4 [LC816950]  Caballeronia-α    ACCAAGGCGACGATCTGTAGCTGGTCTGAGAGGACGACCAGCCACACTGGGACTGAGACA
OTU2 [LC817021]  Caballeronia-β    ACCAAGGCGACGATCTGTAGCTGGTCTGAGAGGACGACCAGCCACACTGGGACTGAGACA
OTU3 [LC816943]  Caballeronia-γ    ACCAAGGCGACGATCTGTAGCTGGTCTGAGAGGACGACCAGCCACACTGGGACTGAGACA
OTU5 [LC816870]  Caballeronia-γ    ACCAAGGCGACGATCTGTAGCTGGTCTGAGAGGACGACCAGCCACACTGGGACTGAGACA
OTU8  [LC817093] Pandoraea         ACCAAGGCGACGATCTGTAGCTGGTCTGAGAGGACGACCAGCCACACTGGGACTGAGACA
OTU9 [LC816937]  Pandoraea         ACCAAGGCGACGATCTGTAGCTGGTCTGAGAGGACGACCAGCCACACTGGGACTGAGACA
OTU10 [LC816935] Pandoraea         ACCAAGGCGACGATCTGTAGCTGGTCTGAGAGGACGACCAGCCACACTGGGACTGAGACA
OTU11 [LC816936] Pandoraea         ACCAAGGCGACGATCTGTAGCTGGTCTGAGAGGACGACCAGCCACACTGGGACTGAGACA
                                   ************************************************************

OTU6 [LC816801]  Paraburkholderia  CGGCCCAGACTCCTACGGGAGGCAGCAGTGGGGAATTTTGGACAATGGGCGCAAGCCTGA
OTU7 [LC816901]  Paraburkholderia  CGGCCCAGACTCCTACGGGAGGCAGCAGTGGGGAATTTTGGACAATGGGCGCAAGCCTGA
OTU1 [LC816990]  Caballeronia-α    CGGCCCAGACTCCTACGGGAGGCAGCAGTGGGGAATTTTGGACAATGGGGGCAACCCTGA
OTU4 [LC816950]  Caballeronia-α    CGGCCCAGACTCCTACGGGAGGCAGCAGTGGGGAATTTTGGACAATGGGGGCAACCCTGA
OTU2 [LC817021]  Caballeronia-β    CGGCCCAGACTCCTACGGGAGGCAGCAGTGGGGAATTTTGGACAATGGGGGAAACCCTGA
OTU3 [LC816943]  Caballeronia-γ    CGGCCCAGACTCCTACGGGAGGCAGCAGTGGGGAATTTTGGACAATGGGGGAAACCCTGA
OTU5 [LC816870]  Caballeronia-γ    CGGCCCAGACTCCTACGGGAGGCAGCAGTGGGGAATTTTGGACAATGGGCGCAAGCCTGA
OTU8  [LC817093] Pandoraea         CGGCCCAGACTCCTACGGGAGGCAGCAGTGGGGAATTTTGGACAATGGGCGAAAGCCTGA
OTU9 [LC816937]  Pandoraea         CGGCCCAGACTCCTACGGGAGGCAGCAGTGGGGAATTTTGGACAATGGGCGAAAGCCTGA
OTU10 [LC816935] Pandoraea         CGGCCCAGACTCCTACGGGAGGCAGCAGTGGGGAATTTTGGACAATGGGCGAAAGCCTGA
OTU11 [LC816936] Pandoraea         CGGCCCAGACTCCTACGGGAGGCAGCAGTGGGGAATTTTGGACAATGGGCGAAAGCCTGA
                                   ************************************************* * ** *****

OTU6 [LC816801]  Paraburkholderia  TCCAGCAATGCCGCGTGTGTGAAGAAGGCCTTCGGGTTGTAAAGCACTTTTGTCCGGAAA
OTU7 [LC816901]  Paraburkholderia  TCCAGCAATGCCGCGTGTGTGAAGAAGGCCTTCGGGTTGTAAAGCACTTTTGTCCGGAAA
OTU1 [LC816990]  Caballeronia-α    TCCAGCAATGCCGCGTGTGTGAAGAAGGCCTTCGGGTTGTAAAGCACTTTTGTCCGGAAA
OTU4 [LC816950]  Caballeronia-α    TCCAGCAATGCCGCGTGTGTGAAGAAGGCCTTCGGGTTGTAAAGCACTTTTGTCCGGAAA
OTU2 [LC817021]  Caballeronia-β    TCCAGCAATGCCGCGTGTGTGAAGAAGGCCTTCGGGTTGTAAAGCACTTTTGTCCGGAAA
OTU3 [LC816943]  Caballeronia-γ    TCCAGCAATGCCGCGTGTGTGAAGAAGGCCTTCGGGTTGTAAAGCACTTTTGTCCGGAAA
OTU5 [LC816870]  Caballeronia-γ    TCCAGCAATGCCGCGTGTGTGAAGAAGGCCTTCGGGTTGTAAAGCACTTTTGTCCGGAAA
OTU8  [LC817093] Pandoraea         TCCAGCAATGCCGCGTGTGTGAAGAAGGCCTTCGGGTTGTAAAGCACTTTTGTCCGGAAA
OTU9 [LC816937]  Pandoraea         TCCAGCAATGCCGCGTGTGTGAAGAAGGCCTTCGGGTTGTAAAGCACTTTTGTCCGGAAA
OTU10 [LC816935] Pandoraea         TCCAGCAATGCCGCGTGTGTGAAGAAGGCCTTCGGGTTGTAAAGCACTTTTGTCCGGAAA
OTU11 [LC816936] Pandoraea         TCCAGCAATGCCGCGTGTGTGAAGAAGGCCTTCGGGTTGTAAAGCACTTTTGTCCGGAAA
                                   ************************************************************
```

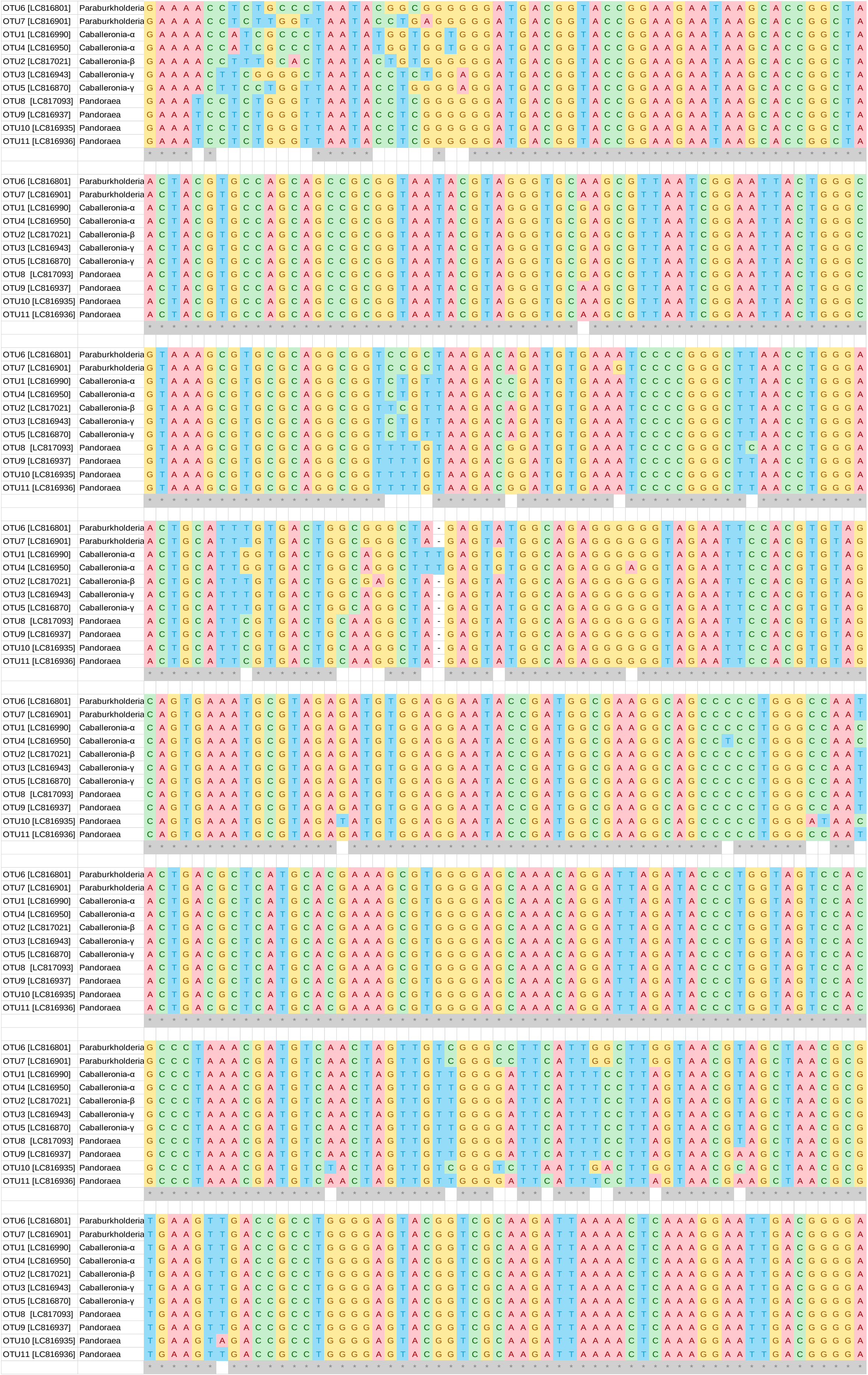

| | | |
|---|---|---|
| OTU6 [LC816801] | Paraburkholderia | CCCGCACAAGC-GGTGGATGATGTGGATTAATTCGATGCAACGCGAAAAACCTTACCTAC |
| OTU7 [LC816901] | Paraburkholderia | CCCGCACAAGC-GGTGGATGATGTGGATTAATTCGATGCAACGCGAAAAACCTTACCTAC |
| OTU1 [LC816990] | Caballeronia-α | CCCGCACAAGCAGGTGGATGATGTGGATTAATTCGATGCAACGCGAAAAACCTTACCTAC |
| OTU4 [LC816950] | Caballeronia-α | CCCGCACAAGC-GGTGGATGATGTGGATTAATTCGATGCAACGCGAAAAACCTTACCTAC |
| OTU2 [LC817021] | Caballeronia-β | CCCGCACAAGC-GGTGGATGATGTGGATTAATTCGATGCAACGCGAAAAACCTTACCTAC |
| OTU3 [LC816943] | Caballeronia-γ | CCCGCACAAGC-GGTGGATGATGTGGATTAATTCGATGCAACGCGAAAAACCTTACCTAC |
| OTU5 [LC816870] | Caballeronia-γ | CCCGCACAAGC-GGTGGATGATGTGGATTAATTCGATGCAACGCGAAAAACCTTACCTAC |
| OTU8 [LC817093] | Pandoraea | CCCGCACAAGC-GGTGGATGATGTGGATTAATTCGATGCAACGCGAAAAACCTTACCTAC |
| OTU9 [LC816937] | Pandoraea | CCCGCACAAGC-GGTGGATGATGTGGATTAATTCGATGCAACGCGAAAAACCTTACCTAC |
| OTU10 [LC816935] | Pandoraea | CCCGCACAAGC-GGTGGATGATGTGGATTAATTCGATGCAACGCGAAAAACCTTACCTAC |
| OTU11 [LC816936] | Pandoraea | CCCGCACAAGC-GGTGGATGATGTGGATTAATTCGATGCAACGCGAAAAACCTTACCTAC |
| | | *********** ************************************************ |

| | | |
|---|---|---|
| OTU6 [LC816801] | Paraburkholderia | CCTTGACATGT-ACGGAATCCTGCTGAGAGGTGGGAGTGCCCGAAAGGGAGCCGTAA-CA |
| OTU7 [LC816901] | Paraburkholderia | CCTTGACGTGT-ACGGAATCCTGCTGAGAGGTGGGAGTGCCCGAAAGGGAGCCATAA-CA |
| OTU1 [LC816990] | Caballeronia-α | CCTTGACATGG-TCGGAATCCCGCTGAGAGGTGGGAGTGCTCGAAAGAGAACCGGCG-CA |
| OTU4 [LC816950] | Caballeronia-α | CCTTGACATGG-TCGGAATCCTGCTGAGAGGCGGGAGTGCTCGAAAGAGAACCGGCG-CA |
| OTU2 [LC817021] | Caballeronia-β | CCTTGACATGGGTCGGAACCCTGCTGAAAGGTGGGGGTGCACGAAAGAGAACCGATA-CA |
| OTU3 [LC816943] | Caballeronia-γ | CCTTGACATGG-TCGGAACCCTGGTGAGAGCTGGGGGTGCTCGAAAGAGAACCGACA-CA |
| OTU5 [LC816870] | Caballeronia-γ | CCTTGACATGG-TCGGAATCCTGCTGAGAGGCGGGAGTGCTCGAAAGAGAACCGGCAGCA |
| OTU8 [LC817093] | Pandoraea | CCTTGACATGT-ACGGAAGTCTGCTGAGAGGCGGATGTGCTCGAAAGAGAACCGTAA-CA |
| OTU9 [LC816937] | Pandoraea | CCTTGACATGT-ACGGAATCTTGCTGAGAGGTGAGAGTGCTCGAAAGAGAACCGTAA-CA |
| OTU10 [LC816935] | Pandoraea | CCTTGACATGT-ACGGAAGGCCGAAGAGATTTGGCTGTGCTCGAAAGAGAACCGTAA-CA |
| OTU11 [LC816936] | Pandoraea | CCTTGACATGT-ACGGAATCTTGCTGAGAGGTGAGAGTGCTCGAAAGAGAACCGTAA-CA |
| | | ******* **   *****    *  ** *   *   **** ****** ** **     ** |

| | | |
|---|---|---|
| OTU6 [LC816801] | Paraburkholderia | CAGGTGCTGCATGGCTGTCGTCAGCTCGTGTCGTGAGATGTTGGGTTAAGTCCCGCAACG |
| OTU7 [LC816901] | Paraburkholderia | CAGGTGCTGCATGGCTGTCGTCAGCTCGTGTCGTGAGATGTTGGGTTAAGTCCCGCAACG |
| OTU1 [LC816990] | Caballeronia-α | CAGGTGCTGCATGGCTGTCGTCAGCTCGTGTCGTGAGATGTTGGGTTAAGTCCCGCAACG |
| OTU4 [LC816950] | Caballeronia-α | CAGGTGCTGCATGGCTGTCGTCAGCTCGTGTCGTGAGATGTTGGGTTAAGTCCCGCAACG |
| OTU2 [LC817021] | Caballeronia-β | CAGGTGCTGCATGGCTGTCGTCAGCTCGTGTCGTGAGATGTTGGGTTAAGTCCCGCAACG |
| OTU3 [LC816943] | Caballeronia-γ | CAGGTGCTGCATGGCTGTCGTCAGCTCGTGTCGTGAGATGTTGGGTTAAGTCCCGCAACG |
| OTU5 [LC816870] | Caballeronia-γ | CAGGTGCTGCATGGCTGTCGTCAGCTCGTGTCGTGAGATGTTGGGTTAAGTCCCGCAACG |
| OTU8 [LC817093] | Pandoraea | CAGGTGCTGCATGGCTGTCGTCAGCTCGTGTCGTGAGATGTTGGGTTAAGTCCCGCAACG |
| OTU9 [LC816937] | Pandoraea | CAGGTGCTGCATGGCTGTCGTCAGCTCGTGTCGTGAGATGTTGGGTTAAGTCCCGCAACG |
| OTU10 [LC816935] | Pandoraea | CAGGTGCTGCATGGCTGTCGTCAGCTCGTGTCGTGAGATGTTGGGTTAAGTCCCGCAACG |
| OTU11 [LC816936] | Pandoraea | CAGGTGCTGCATGGCTGTCGTCAGCTCGTGTCGTGAGATGTTGGGTTAAGTCCCGCAACG |
| | | ************************************************************ |

| | | |
|---|---|---|
| OTU6 [LC816801] | Paraburkholderia | AGCGCAACCCTTGTCCCTAGTTGCTACGCAAGAGCACTCCAGGGAGACTGCCGGTGACAA |
| OTU7 [LC816901] | Paraburkholderia | AGCGCAACCCTTGTCCCTAGTTGCTACGCAAGAGCACTCTAGGGAGACTGCCGGTGACAA |
| OTU1 [LC816990] | Caballeronia-α | AGCGCAACCCTTGTCCTTAGTTGCTACGCAAGAGCACTCTAAGGAGACTGCCGGTGACAA |
| OTU4 [LC816950] | Caballeronia-α | AGCGCAACCCTTGTCCTTAGTTGCTACGCAAGAGCACTCTAAGGAGACTGCCGGTGACAA |
| OTU2 [LC817021] | Caballeronia-β | AGCGCAACCCTTGTCCTTAGTTGCTACGCAAGAGCACTCTAAGGAGACTGCCGGTGACAA |
| OTU3 [LC816943] | Caballeronia-γ | AGCGCAACCCTTGTCCTTAGTTGCTACGCAAGAGCACTCTAAGGAGACTGCCGGTGACAA |
| OTU5 [LC816870] | Caballeronia-γ | AGCGCAACCCTTGTCCTTAGTTGCTACGCAAGAGCACTCTAAGGAGACTGCCGGTGACAA |
| OTU8 [LC817093] | Pandoraea | AGCGCAACCCTTGTCCTTAGTTGCTACGCAAGAGCACTCTAAGGAGACTGCCGGTGACAA |
| OTU9 [LC816937] | Pandoraea | AGCGCAACCCTTGTCCTTAGTTGCTACGCAAGAGCACTCTAAGGAGACTGCCGGTGACAA |
| OTU10 [LC816935] | Pandoraea | AGCGCAACCCTTGTCATTAGTTGCTACGAAAGGGCACTCTAATGAGACTGCCGGTGACAA |
| OTU11 [LC816936] | Pandoraea | AGCGCAACCCTTGTCATTAGTTGCTACGAGAGGGCACTCTAATGAGACTGCCGGTGACAA |
| | | ***************  ***********  ** ****** *  ***************** |

| | | |
|---|---|---|
| OTU6 [LC816801] | Paraburkholderia | ACCGGAGGAAGGTGGGGATGACGTCAAGTCCTC-ATGGCCCTTATGGGTAGGGCTT-CAC |
| OTU7 [LC816901] | Paraburkholderia | ACCGGAGGAAGGTGGGGATGACGTCAAGTCCTC-ATGGCCCTTATGGGTAGGGCTT-CAC |
| OTU1 [LC816990] | Caballeronia-α | ACCGGAGGAAGGTGGGGATGACGTCAAGTCCTC-ATGGCCCTTATGGGTAGGGCTT-CAC |
| OTU4 [LC816950] | Caballeronia-α | ACCGGAGGAAGGTGGGGATGACGTCAAGTCCTC-ATGGCCCTTATGGGTAGGGCTT-CAC |
| OTU2 [LC817021] | Caballeronia-β | ACCGGAGGAAGGTGGGGATGACGTCAAGTCCTC-ATGGCCCTTATGGGTAGGGCTT-CAC |
| OTU3 [LC816943] | Caballeronia-γ | ACCGGAGGAAGGTGGGGATGACGTCAAGTCCTC-ATGGCCCTTATGGGTAGGGCTT-CAC |
| OTU5 [LC816870] | Caballeronia-γ | ACCGGAGGAAGGTGGGGATGACGTCAAGTCCTC-ATGGCCCTTATGGGTAGGGCTT-CAC |
| OTU8 [LC817093] | Pandoraea | ACCGGAGGAAGGTGGGGATGACGTCAAGTCCTC-ATGGCCCTTATGGGTAGGGCTT-CAC |
| OTU9 [LC816937] | Pandoraea | ACCGGAGGAAGGTGGGGATGACGTACAGTCCTCCATGGCCCTTATGGGTAGGGCTTACAC |
| OTU10 [LC816935] | Pandoraea | ACCGGAGGAAGGTGGGGATGACGTCAAGTCCTC-ATGGCCCTTATGGGTAGGGCTT-CAC |
| OTU11 [LC816936] | Pandoraea | ACCGGAGGAAGGTGGGGATGACGTCAAGTCCTC-ATGGCCCTTATGGGTAGGGCTT-CAC |
| | | ************************  ******* ********************** *** |

| | | |
|---|---|---|
| OTU6 [LC816801] | Paraburkholderia | ACGTCATACAATGGTCGGAACAGAGGGTCGCCAAGCCGCGAGGTGGAGCCAATCCCAGAA |
| OTU7 [LC816901] | Paraburkholderia | ACGTCATACAATGGTCGGAACAGAGGGTCGCCAACCCGCGAGGGGGAGCCAATCCCAGAA |
| OTU1 [LC816990] | Caballeronia-α | ACGTCATACAATGGTCGGAACAGAGGGTTGCCAAGCCGCGAGGTGGAGCCAATCCCAGAA |
| OTU4 [LC816950] | Caballeronia-α | ACGTCATACAATGGTCGGAACAGAGGGTTGCCAAGCCGCGAGGCGGAGCCAATCCCAGAA |
| OTU2 [LC817021] | Caballeronia-β | ACGTCATACAATGGTCGGAACAGAGGGCTGCCAACCCGTGAGGGGGAGCTAATCCCAGAA |
| OTU3 [LC816943] | Caballeronia-γ | ACGTCATACAATGGTCGGAACAGAGGGTCGCTAAGCCGCGAGGTGGAGCCAATCCCAGAA |
| OTU5 [LC816870] | Caballeronia-γ | ACGTCATACAATGGTCGGAACAGAGGGTCGCTAAGCCGCGAGGTGGAGCCAATCCCAGAA |
| OTU8 [LC817093] | Pandoraea | ACGTCATACAATGGTCGGTACAGAGGGCTGCCAAACCGCGAGGTGGAGCTAACCCCAGAA |
| OTU9 [LC816937] | Pandoraea | ACGTCATACAATGGTCGGTACAGAGGGCTGCCAAACCGCGAGGTGGAGCTAACCCCAGAA |
| OTU10 [LC816935] | Pandoraea | ACGTCATACAATGGTACATACAGAGGGCCGCCAACCCGCGAGGGGGAGCTAATCCCAGAA |
| OTU11 [LC816936] | Pandoraea | ACGTCATACAATGGTACATACAGAGGGCCGCCAACCCGCGAGGGGGTGCTAATCCCAGAA |
| | | ***************    ********  ** ** *** **** ** ** ** ******* |

| | | |
|---|---|---|
| OTU6 [LC816801] | Paraburkholderia | AACCGATCGTAGTCCGGATCGCACTCTGCAACTCGAGTGCGTGAAGCTGGAATCGCTAGT |
| OTU7 [LC816901] | Paraburkholderia | AACCGATCGTAGTCCGGATCGCACTCTGCAACTCGAGTGCGTGAAGCTGGAATCGCTAGT |
| OTU1 [LC816990] | Caballeronia-α | AACCGATCGTAGTCCGGATCGCAGTCTGCAACTCGACTGCGTGAAGCTGGAATCGCTAGT |
| OTU4 [LC816950] | Caballeronia-α | AACCGATCGTAGTCCGGATCGCAGTCTGCAACTCGACTGCGTGAAGCTGGAATCGCTAGT |
| OTU2 [LC817021] | Caballeronia-β | AACCGATCGTAGTCCGGATCGTAGTCTGCAACTCGACTACGTGAAGCTGGAATCGCTAGT |
| OTU3 [LC816943] | Caballeronia-γ | AACCGATCGTAGTCCGGATCGTAGTCTGCAACTCGACTACGTGAAGCTGGAATCGCTAGT |
| OTU5 [LC816870] | Caballeronia-γ | AACCGATCGTAGTCCGGATCGCAGTCTGCAACTCGACTGCGTGAAGCTGGAATCGCTAGT |
| OTU8 [LC817093] | Pandoraea | AACCGATCGTAGTCCGGATCGCAGTCTGCAACTCGACTGCGTGAAGCTGGAATCGCTAGT |
| OTU9 [LC816937] | Pandoraea | AACCGATCGTAGTCCGGATCGCAGTCTGCAACTCGACTGCGTGAAGCTGGAATCGCTAGT |
| OTU10 [LC816935] | Pandoraea | AGTGTATCGTAGTCCGGATTGTAGTCTGCAACTCGACTACATGAAGTTGGAATCGCTAGT |
| OTU11 [LC816936] | Pandoraea | AGTGTATCGTAGTCCGGATTGTAGTCTGCAACTCGACTACATGAAGTTGGAATCGCTAGT |
| | | *    ************** * * ************ * * ***** ************* |

| | | |
|---|---|---|
| OTU6 [LC816801] | Paraburkholderia | AATCGCGGATCAGCATGCCGCGGTGAATACGTTCCCGGGTCTTGTACACACCGCCCGTCA |
| OTU7 [LC816901] | Paraburkholderia | AATCGCGGATCAGCATGCCGCGGTGAATACGTTCCCGGGTCTTGTGCACACCGCCCGTCA |
| OTU1 [LC816990] | Caballeronia-α | AATCGCGGATCAGCATGCCGCGGTGAATACGTTCCCGGGTCTTGTACACACCGCCCGTCA |
| OTU4 [LC816950] | Caballeronia-α | AATCGCGGATCAGCATGCCGCGGTGAATACGTTCCCGGGTCTTGTACACACCGCCCGTCA |
| OTU2 [LC817021] | Caballeronia-β | AATCGCGGATCAGCATGCCGCGGTGAATACGTTCCCGGGTCTTGTACACACCGCCCGTCA |
| OTU3 [LC816943] | Caballeronia-γ | AATCGCGGATCAGCATGCCGCGGTGAATACGTTCCCGGGTCTTGTACACACCGCCCGTCA |
| OTU5 [LC816870] | Caballeronia-γ | AATCGCGGATCAGCATGCCGCGGTGAATACGTTCCCGGGTCTTGTACACACCGCCCGTCA |
| OTU8 [LC817093] | Pandoraea | AATCGCGGATCAGCATGTCGCGGTGAATACGTTCCCGGGTCTTGTACACACCGCCCGTCA |
| OTU9 [LC816937] | Pandoraea | AATCGCGGATCAGCATGTCGCGGTGAATACGTTCCCGGGTCTTGTACACACCGCCCGTCA |
| OTU10 [LC816935] | Pandoraea | AATCGCGGATCAGCATGTCGCGGTGAATACGTTCCCGGGTCTTGTACACACCGCCCGTCA |
| OTU11 [LC816936] | Pandoraea | AATCGCGGATCAGCATGTCGCGGTGAATACGTTCCCGGGTCTTGTACACACCGCCCGTCA |
| | | ***************** *************************** ************** |

```
OTU6 [LC816801]  Paraburkholderia  CACCAT-GGGAGTGGGTTTTACCAGAAGTGGCTAGTCTAAC-CGCAAGGAGGACGGTCAC
OTU7 [LC816901]  Paraburkholderia  CACCAT-GGGAGTGGGTTTTACCAGAAGTGGCTAGCCTAAC-CGCAAGGAGGACGGTCAC
OTU1 [LC816990]  Caballeronia-α    CACCAT-GGGAGTGGGTTTCACCAGAAGTAGGTAGCCTAAC-CGCAAGGAGGGCGCTTAC
OTU4 [LC816950]  Caballeronia-α    CACCAT-GGGAGTGGGTTTTACCAGAAGTGGCTAGTCTAAC-CGCAAGGAGGACGGTCAC
OTU2 [LC817021]  Caballeronia-β    CACCAT-GGGAGTGGGTTTCACCAGAAGTAGGTAGCCTAAC-CGCAAGGAGGGCGCTTAC
OTU3 [LC816943]  Caballeronia-γ    CACCATCGGGAGTGGGTTTTACCAGAAGTCGCTAGTCTAACGCGCAAGGAGGACGGTCAC
OTU5 [LC816870]  Caballeronia-γ    CACCAT-GGGAGTGGGTTTTACCAGAAGTGGCTAGTCTAAC-CGCAAGGAGGACGGTCAC
OTU8  [LC817093] Pandoraea         CACCAT-GGGAGTGGGTTTTGCCAGAAGTAGGTAGCCTAAC-CGTAAGGAGGGCGCTTAC
OTU9 [LC816937]  Pandoraea         CACCAT-GGGAGTGGGTTTTGCCAGAAGTAGGTAGCCTAAC-CGTAAGGAGGGCGCTTAC
OTU10 [LC816935] Pandoraea         CACCAT-GGGAGCGGGTTCTGCCAGAAGTAGTTAGCCTAAC-CGCAAGGAGGGCGATTAC
OTU11 [LC816936] Pandoraea         CACCAT-GGGAGCGGGTTCTGCCAGAAGTAGTTAGCCTAAC-CGCAAGGAGGGCGATTAC
                                   ****** ***** *****   ******** * *** ***** ** ******* ** * **

OTU6 [LC816801]  Paraburkholderia  CACGGTAGGATTCATGACTGGGGTGAAGTCGTAACAAGGTAGCCGTATCGGAAGG
OTU7 [LC816901]  Paraburkholderia  CACGGTAGGATTCATGACTGGGGTGAAGTCGTAACAAGGTAGCCGTATCGGAAGG
OTU1 [LC816990]  Caballeronia-α    CACGGTGGGATTCATGACTGGGGTGAAGTCGTAACAAGGTAGCCGTATCGGAAGG
OTU4 [LC816950]  Caballeronia-α    CACGGTAGGATTCATGACTGGGGTGAAGTCGTAACAAGGTAGCCGTATCGGAAGG
OTU2 [LC817021]  Caballeronia-β    CACGGTGGGATTCATGACTGGGGTGAAGTCGTAACAAGGTAGCCGTATCGGAAGG
OTU3 [LC816943]  Caballeronia-γ    CACGGTAGGATTCATGACTGGGGTGAAGTCGTAACAAGGTAGCCGTATCGGAAGG
OTU5 [LC816870]  Caballeronia-γ    CACGGTAGGATTCATGACTGGGGTGAAGTCGTAACAAGGTAGCCGTATCGGAAGG
OTU8  [LC817093] Pandoraea         CACGGCAGGATTCATGACTGGGGTGAAGTCGTAACAAGGTAGCCGTAGGGGAACC
OTU9 [LC816937]  Pandoraea         CACGGCAGGATTCATGACTGGGGTGAAGTCGTAACAAGGTAGCCGTAGGGGAACC
OTU10 [LC816935] Pandoraea         CACGGCAGGGTTCGTGACTGGGGTGAAGTCGTAACAAGGTAGCCGTATCGGAAGG
OTU11 [LC816936] Pandoraea         CACGGCAGGGTTCGTGACTGGGGTGAAGTCGTAACAAGGTAGCCGTATCGGAAGG
                                   *****  ** *** ********************************** **** 
```

**Figure S1. Alignment of OTUs identified in the midgut crypts of *Riptortus pedestris* and *Coreus marginatus*.** The sequences of 16S rRNA gene PCR products were aligned with the MAFFT program. An asterisk indicates a correspondence of sequences for all OTUs.

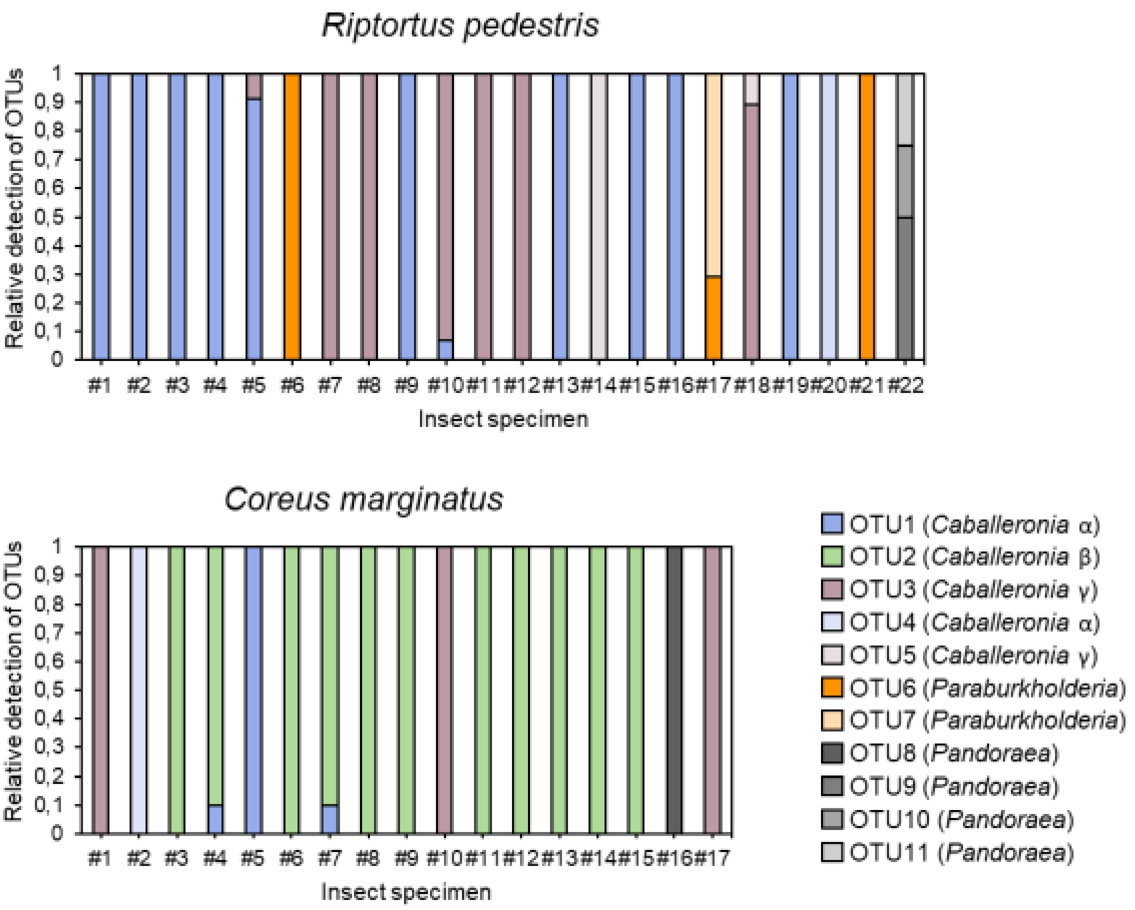


**Figure S2. Distribution of OTUs of the 16S rRNA gene in individual *Riptortus pedestris* and *Coreus marginatus* M4 midgut samples.** Bar plots representing the relative proportion of OTUs detected in each M4 midgut sample from *R. pedestris* and *C. marginatus* individuals reared on the same soil sample (see Table S2 for data).

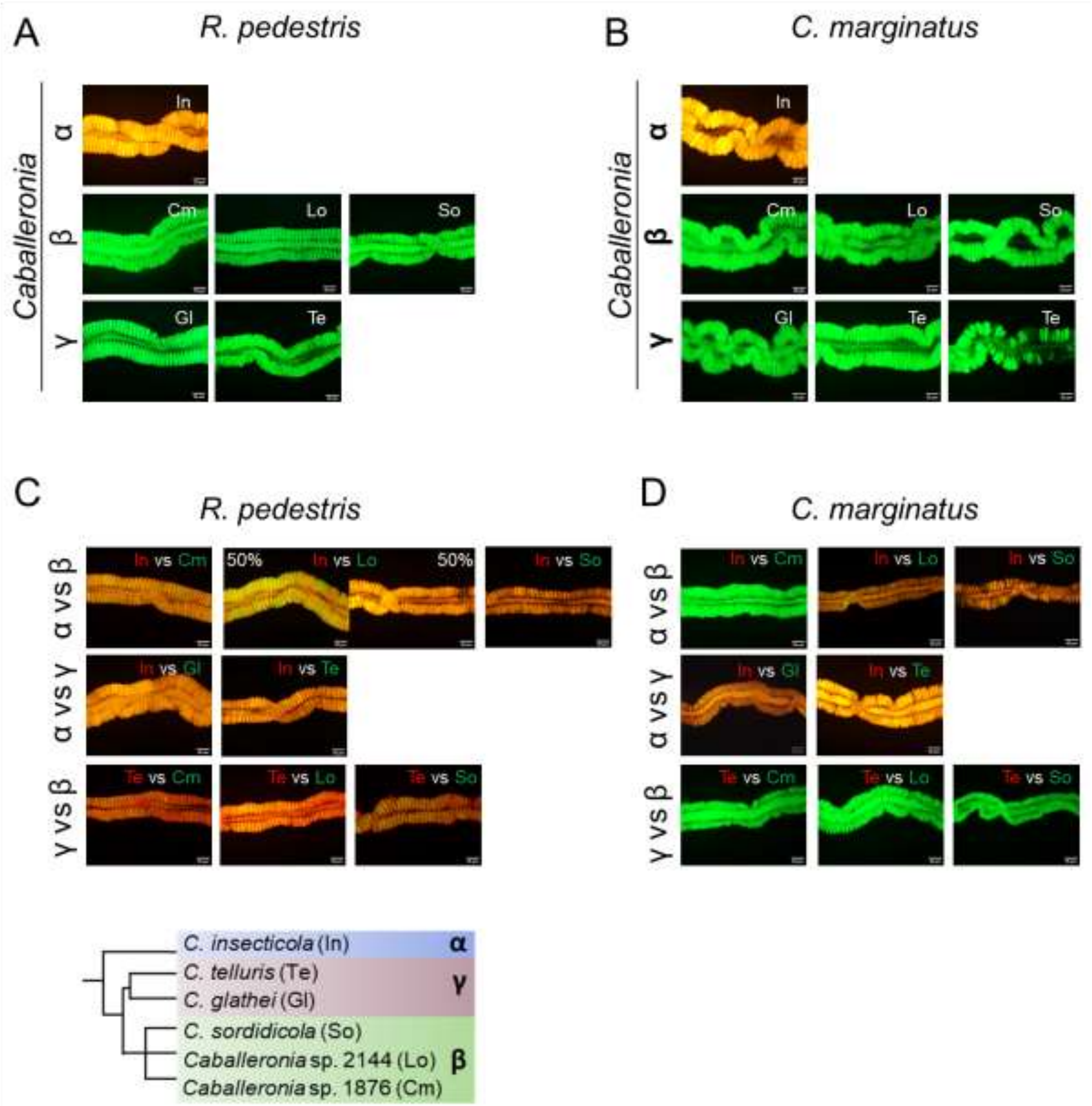


**Figure S3. The colonization ability of *Caballeronia* species in the midgut of *Riptortus pedestris* and *Coreus marginatus*. A.** Representative micrographs of midguts of *R. pedestris*, infected with a single *Caballeronia* strain and dissected at 5 dpi. Scale bars are 30 µm. **B.** Representative micrographs of midgut crypts of *C. marginatus*, infected with a single *Caballeronia* strain and dissected at 7 dpi. The crypt region of individuals infected with *C. telluris* (Te) is either fully colonized (as in left Te picture) or in some cases only partially colonized (as in right Te picture). **C.** Representative micrographs of midguts of *R. pedestris*, co-infected with two *Caballeronia* strains and dissected at 5 dpi. **D.** Representative micrographs of midgut crypts of *C. marginatus*, co-infected with two *Caballeronia* strains and dissected at 7 dpi. Scale bars are 40 µm.

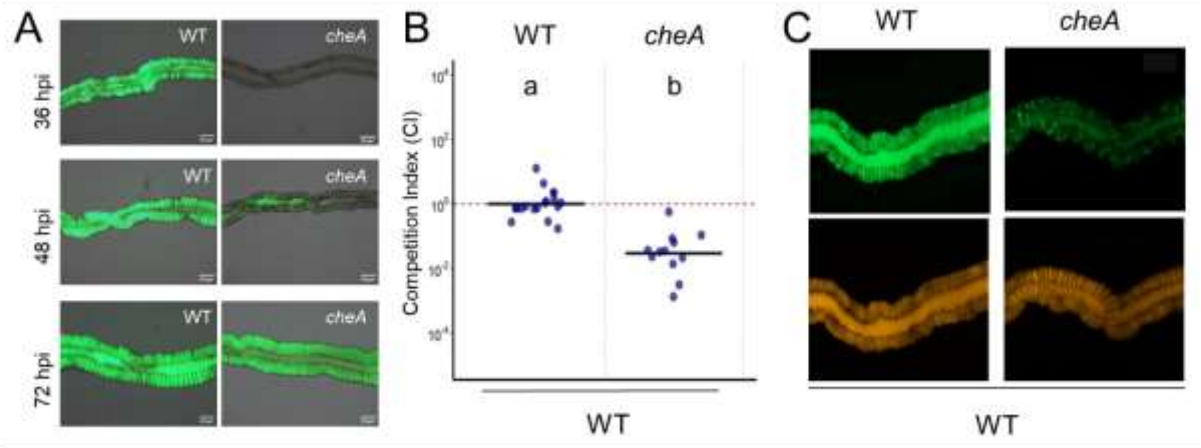


**Figure S4. The colonization ability of the *Caballeronia cheA* mutant in the *Riptortus pedestris* midgut. A**. Microscopy image of the M4 colonization at 36, 48 and 72 hpi of R. *pedestris* with the wild-type strain (WT) and the *cheA* mutant ($n = 10$), showing the delayed colonization dynamics by the *cheA* mutant compared to the wild-type strain. **B**. Competition index of *Caballeronia* mutant *cheA* with the wild-type strain in *R. pedestris* ($n > 10$). Letters indicate significant differences ($P < 0.05$) using the Kruskal-Wallis test with the post-hoc pairwise Wilcoxon rank with the Benjamini-Hochberg *P*-value adjustment method. **C.** Microscopy images of pairwise competitions between GFP- and mScarlet-I-labelled *C. insecticola* strains.

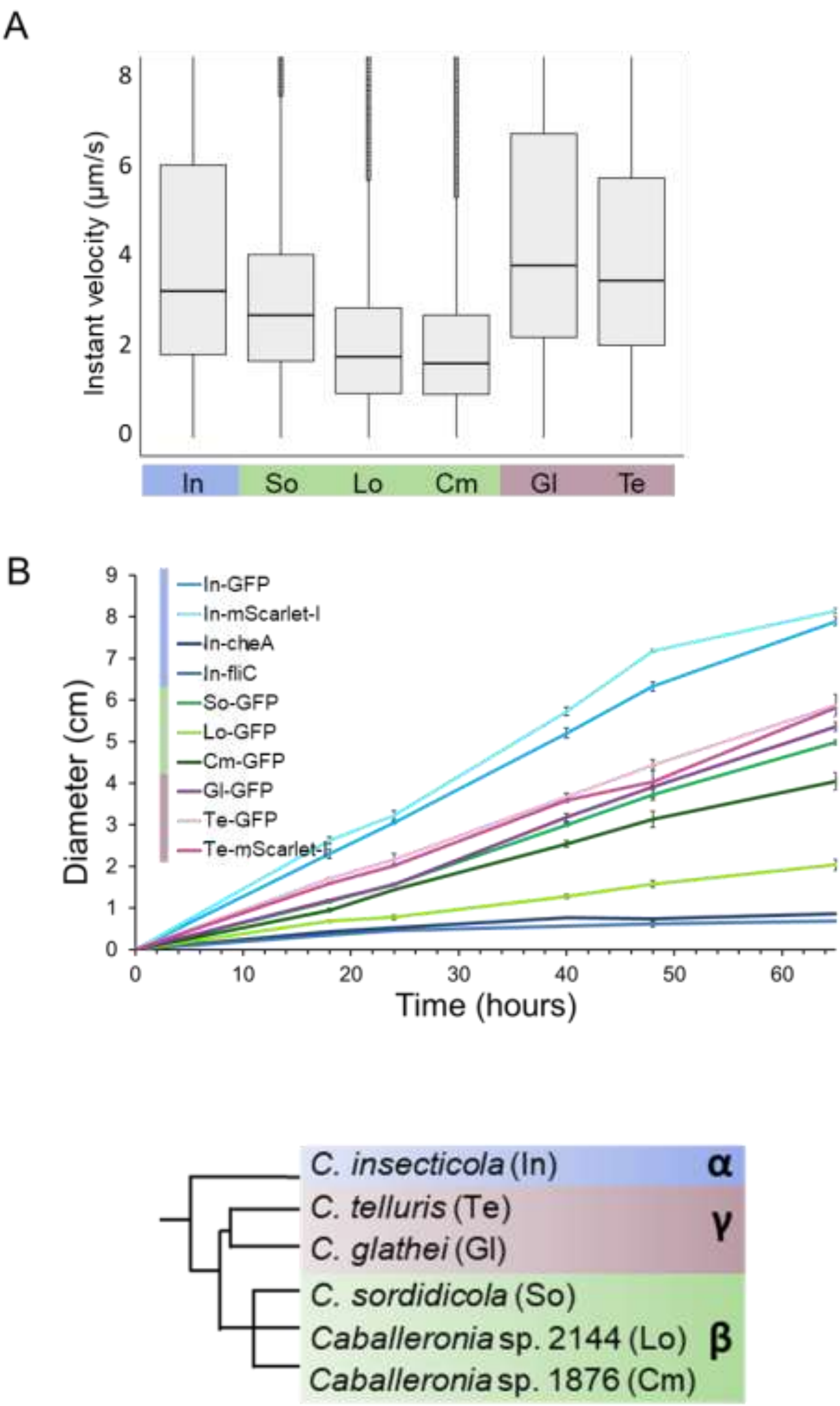


**Figure S5. Motility and chemotaxis of *Caballeronia* species. A.** Box plots representing the velocity of strains in liquid YG medium. Box limits indicate the range of the central 50% of the data, the black line within the box indicates the median of the data set, and the thick black lines running above the whiskers represent the outlier values. **B.** The combined motility and chemotaxis of strains measured on 0.3% YG agar swimming plates. The graphs shows the diameter of growth on the plates in function of time. The used strains are abbreviated according to the key at the bottom.

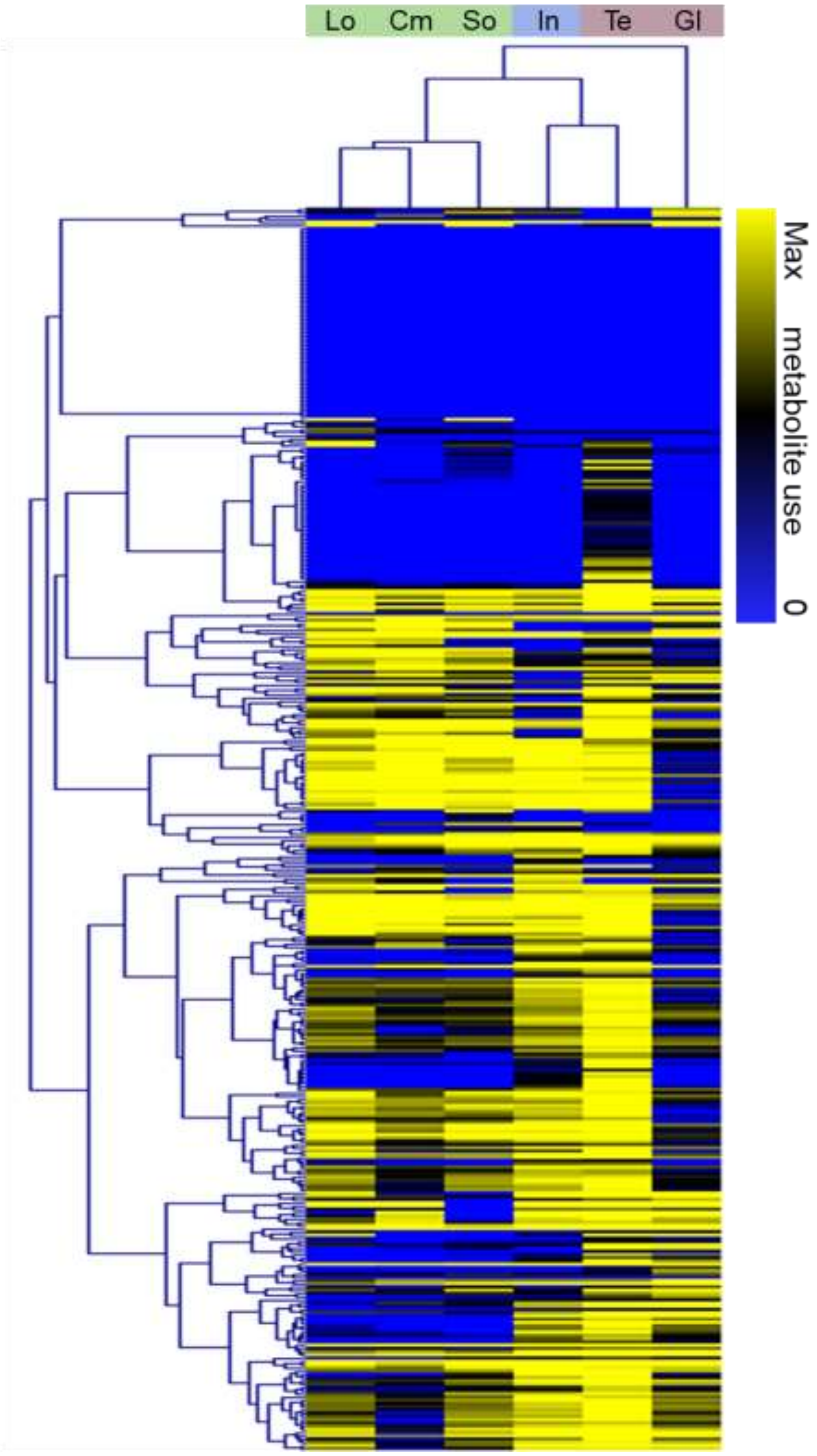


**Figure S6. Biolog phenotyping of *Caballeronia* strains.** The heat map shows metabolite usage by *Caballeronia* strains for the combined 486 tested conditions by Biolog plates PM1, PM2A, PM3B and PM4A. Data represents areas under the absorbance curves in function of time, normalized to 1 relative to the maximal value in each microplate. Metabolites and strains were ordered with Pearson correlation using hierarchical cluster analysis. The compound order in is available in the Supplementary Data Set 1.

## SUPPLEMENTARY TABLES

| Strain | Description | Reference |
|---|---|---|
| *Caballeronia insecticola* RPE75 (In) | rifampicin-resistant derivative of wild type strain RPE64, isolated from *Riptortus pedestris* , japan | 8 |
| *Caballeronia insecticola* RPE225 (In-GFP) | Tn*7* -GFP derivative of RPE75 | 2 |
| *Caballeronia insecticola* Scarlet (In-mScarlet-I) | Tn*7* -mScarlet-I derivative of RPE75 | 1 |
| Cm1876 (Cm) | strain isolate of *Coreus marginatus* , France | 5 |
| Cm1876 (Cm-GFP) | Tn*7* -GFP derivative of the strain Cm1876 | This work |
| *Caballeronia sordidicola* (So) | Type strain DSM17212$^{T}$; isolated from a culture of the fungus Phanerochaete sordida, South Korea | Type strain |
| *Caballeronia sordidicola* (So-GFP) | Tn*7* -GFP derivative of *C. sordidicola* | This work |
| Lo2144 (Lo) | Strain isolate of *Leptoglossus occidentalis* , France | 6 |
| Lo2144 (Lo-GFP) | Tn*7* -GFP derivative of strain Lo2144 | This work |
| *Caballeronia glathei* (Gl) | Type strain JCM10563$^{T}$, isolated from soil, Germany | Type strain |
| *Caballeronia glathei* (Gl-GFP) | Tn*7* -GFP derivative of *C. glathei* | This work |
| *Caballeronia telluris* (Te) | Type strain LMG22936$^{T}$, isolated from soil, Netherlands | Type strain |
| *Caballeronia telluris* (Te-GFP) | Tn*7* -GFP derivative of *C. telluris* | This work |
| *Caballeronia telluris* (Te-mScarlet-I) | Tn*7* -mScarlet-I derivative of *C. telluris* | This work |
| *Caballeronia insecticola* RPE75.Δ*wzm* | *wzm* (BRPE64_RS10560) deletion mutant | 3 |
| *Caballeronia insecticola* RPE75.Δ*tpr* | *tpr (BRPE64_RS10075)* deletion mutant | 3 |
| *Caballeronia insecticola* RPE75.*cheA::* Tn5 | *cheA(BRPE64_RS19435)* Tn*5* transposon insertion mutant | 4 |
| *Caballeronia insecticola* RPE75.Δ*fbp* | *fbp (BRPE64_RS03750)* deletion mutant | 1 |
| *Caballeronia insecticola* RPE75.Δ*pps* | *pps (BRPE64_RS05810)* deletion mutant | 1 |
| WM3064.pURR25 | *E. coli* carring Tn*7* -GFP on plasmid | 9 |
| WM3064.pUX-BF13 | *E. coli* helper carring transposase | 9 |
| S17-1λpir.pMRE-Tn*7* -135 | *E. coli* carring Tn*7* -mScarlett-I on plasmid | 7 |

**Table S1. Bacterial strains used in this study.**


1. Jouan R, Lextrait G, Lachat J *et al.* Transposon sequencing reveals the essential gene set and genes enabling gut symbiosis in the insect symbiont *Caballeronia insecticola*. *ISME Commun* 2024;**4**:ycad001. https://doi.org/10.1093/ismeco/ycad001
2. Kikuchi Y, Fukatsu T. Live imaging of symbiosis: spatiotemporal infection dynamics of a GFP-labelled *Burkholderia* symbiont in the bean bug *Riptortus pedestris*. *Mol Ecol* 2014;**23**:1445-56. https://doi.org/10.1111/mec.12479
3. Lachat J, Lextrait G, Jouan R *et al.* Hundreds of antimicrobial peptides create a selective barrier for insect gut symbionts. *Proc Natl Acad Sci U S A* 2024;**121**:e2401802121. https://doi.org/10.1073/pnas.2401802121
4. Ohbayashi T, Takeshita K, Kitagawa W *et al.* Insect's intestinal organ for symbiont sorting. *Proc Natl Acad Sci U S A* 2015;**112**:E5179-88. https://doi.org/10.1073/pnas.1511454112
5. Ohbayashi T, Itoh H, Lachat J *et al. Burkholderia* gut symbionts associated with European and Japanese populations of the dock bug *Coreus marginatus* (Coreoidea: Coreidae). *Microbes Environ* 2019;**34**:219-22. https://doi.org/10.1264/jsme2.ME19011
6. Ohbayashi T, Cossard R, Lextrait G *et al.* Intercontinental diversity of *Caballeronia* gut symbionts in the conifer pest bug *Leptoglossus occidentalis*. *Microbes Environ* 2022;**37**:ME22042. https://doi.org/10.1264/jsme2.ME22042
7. Schlechter RO, Jun H, Bernach M *et al.* Chromatic bacteria - a broad host-range plasmid and chromosomal insertion toolbox for fluorescent protein expression in bacteria. *Front Microbiol* 2018;**9**:3052. https://doi.org/10.3389/fmicb.2018.03052
8. Takeshita K, Tamaki H, Ohbayashi T *et al. Burkholderia insecticola* sp. nov., a gut symbiotic bacterium of the bean bug *Riptortus pedestris*. *Int J Syst Evol Microbiol* 2018;**68**:2370-4. https://doi.org/10.1099/ijsem.0.002848
9. Teal TK, Lies DP, Wold BJ, Newman DK. Spatiometabolic stratification of *Shewanella oneidensis* biofilms. *Appl Environ Microbiol* 2006;**72**:7324-30. https://doi.org/10.1128/AEM.01163-06

| Sample name | Individual No. | Caballeronia α OTU1 | Caballeronia β OTU2 | Caballeronia γ OTU3 | Caballeronia α OTU4 | Caballeronia γ OTU5 | Paraburkholderia OTU6 | Paraburkholderia OTU7 | Pandoraea OTU8 | Pandoraea OTU9 | Pandoraea OTU10 | Pandoraea OTU11 | Total |
|---|---|---|---|---|---|---|---|---|---|---|---|---|---|
| ***R. pedestris*** | | | | | | | | | | | | | |
| R7 | #1 | 1 | 0 | 0 | 0 | 0 | 0 | 0 | 0 | 0 | 0 | 0 | 14 |
| R11 | #2 | 1 | 0 | 0 | 0 | 0 | 0 | 0 | 0 | 0 | 0 | 0 | 13 |
| R15 | #3 | 1 | 0 | 0 | 0 | 0 | 0 | 0 | 0 | 0 | 0 | 0 | 13 |
| R16 | #4 | 1 | 0 | 0 | 0 | 0 | 0 | 0 | 0 | 0 | 0 | 0 | 11 |
| R17 | #5 | 0,91 | 0 | 0,09 | 0 | 0 | 0 | 0 | 0 | 0 | 0 | 0 | 11 |
| R18 | #6 | 0 | 0 | 0 | 0 | 0 | 1 | 0 | 0 | 0 | 0 | 0 | 4 |
| R20 | #7 | 0 | 0 | 1 | 0 | 0 | 0 | 0 | 0 | 0 | 0 | 0 | 4 |
| R25 | #8 | 0 | 0 | 1 | 0 | 0 | 0 | 0 | 0 | 0 | 0 | 0 | 6 |
| R26 | #9 | 1 | 0 | 0 | 0 | 0 | 0 | 0 | 0 | 0 | 0 | 0 | 3 |
| R27 | #10 | 0,07 | 0 | 0,93 | 0 | 0 | 0 | 0 | 0 | 0 | 0 | 0 | 15 |
| R28 | #11 | 0 | 0 | 1 | 0 | 0 | 0 | 0 | 0 | 0 | 0 | 0 | 6 |
| R29 | #12 | 0 | 0 | 1 | 0 | 0 | 0 | 0 | 0 | 0 | 0 | 0 | 14 |
| R31 | #13 | 1 | 0 | 0 | 0 | 0 | 0 | 0 | 0 | 0 | 0 | 0 | 10 |
| R32 | #14 | 0 | 0 | 0 | 0 | 1 | 0 | 0 | 0 | 0 | 0 | 0 | 11 |
| R33 | #15 | 1 | 0 | 0 | 0 | 0 | 0 | 0 | 0 | 0 | 0 | 0 | 13 |
| R37 | #16 | 1 | 0 | 0 | 0 | 0 | 0 | 0 | 0 | 0 | 0 | 0 | 13 |
| R38 | #17 | 0 | 0 | 0 | 0 | 0 | 0,29 | 0,71 | 0 | 0 | 0 | 0 | 14 |
| R39 | #18 | 0 | 0 | 0,89 | 0 | 0,11 | 0 | 0 | 0 | 0 | 0 | 0 | 9 |
| R40 | #19 | 1 | 0 | 0 | 0 | 0 | 0 | 0 | 0 | 0 | 0 | 0 | 4 |
| R41 | #20 | 0 | 0 | 0 | 1 | 0 | 0 | 0 | 0 | 0 | 0 | 0 | 5 |
| R42 | #21 | 0 | 0 | 0 | 0 | 0 | 1 | 0 | 0 | 0 | 0 | 0 | 3 |
| R43 | #22 | 0 | 0 | 0 | 0 | 0 | 0 | 0 | 0 | 0,5 | 0,25 | 0,25 | 4 |
| | Rel. Total | 0,53 | 0,00 | 0,27 | 0,03 | 0,06 | 0,06 | 0,05 | 0,00 | 0,01 | 0,01 | 0,01 | |
| | Abs. Total | 105 | 0 | 53 | 5 | 12 | 11 | 10 | 0 | 2 | 1 | 1 | 200 |
| ***C. marginatus*** | | | | | | | | | | | | | |
| C3 | #1 | 0 | 0 | 1 | 0 | 0 | 0 | 0 | 0 | 0 | 0 | 0 | 11 |
| C4 | #2 | 0 | 0 | 0 | 1 | 0 | 0 | 0 | 0 | 0 | 0 | 0 | 12 |
| C6 | #3 | 0 | 1 | 0 | 0 | 0 | 0 | 0 | 0 | 0 | 0 | 0 | 10 |
| C7 | #4 | 0,1 | 0,9 | 0 | 0 | 0 | 0 | 0 | 0 | 0 | 0 | 0 | 10 |
| C8 | #5 | 1 | 0 | 0 | 0 | 0 | 0 | 0 | 0 | 0 | 0 | 0 | 13 |
| C13 | #6 | 0 | 1 | 0 | 0 | 0 | 0 | 0 | 0 | 0 | 0 | 0 | 9 |
| C30 | #7 | 0,1 | 0,9 | 0 | 0 | 0 | 0 | 0 | 0 | 0 | 0 | 0 | 10 |
| C31 | #8 | 0 | 1 | 0 | 0 | 0 | 0 | 0 | 0 | 0 | 0 | 0 | 12 |
| C32 | #9 | 0 | 1 | 0 | 0 | 0 | 0 | 0 | 0 | 0 | 0 | 0 | 12 |
| C33 | #10 | 0 | 0 | 1 | 0 | 0 | 0 | 0 | 0 | 0 | 0 | 0 | 16 |
| C34 | #11 | 0 | 1 | 0 | 0 | 0 | 0 | 0 | 0 | 0 | 0 | 0 | 5 |
| C35 | #12 | 0 | 1 | 0 | 0 | 0 | 0 | 0 | 0 | 0 | 0 | 0 | 18 |
| C36 | #13 | 0 | 1 | 0 | 0 | 0 | 0 | 0 | 0 | 0 | 0 | 0 | 6 |
| C37 | #14 | 0 | 1 | 0 | 0 | 0 | 0 | 0 | 0 | 0 | 0 | 0 | 5 |
| C38 | #15 | 0 | 1 | 0 | 0 | 0 | 0 | 0 | 0 | 0 | 0 | 0 | 5 |
| C39 | #16 | 0 | 0 | 0 | 0 | 0 | 0 | 0 | 1 | 0 | 0 | 0 | 4 |
| C40 | #17 | 0 | 0 | 1 | 0 | 0 | 0 | 0 | 0 | 0 | 0 | 0 | 5 |
| | Rel. Total | 0,09 | 0,61 | 0,20 | 0,07 | 0,00 | 0,00 | 0,00 | 0,02 | 0,00 | 0,00 | 0,00 | |
| | Abs. Total | 15 | 100 | 32 | 12 | 0 | 0 | 0 | 4 | 0 | 0 | 0 | 163 |

**Table S2. Distribution of OTUs of the 16S rRNA gene in *Riptortus pedestris* and *Coreus marginatus* M4 midgut samples.** The numbers in the OTU columns indicate the proportion of clones belonging to each OTU per insect sample. The figures in the Rel. Total row indicate the proportion of clones belonging to each OTU per insect, while the figures in the Abs. Total row indicate the total number of sequences for each OTU per insect. The figures in the "Total" column indicate the number of sequences obtained per insect and in total for all the insects per species.

## SUPPLEMENTARY MATERIALS AND METHODS

### Insects and bacterial strains

The *R. pedestris* TKS1 inbred line used in this study is derived from a single male and female pair that were collected in 2007, from a soybean field in Tsukuba, Ibaraki, Japan. *C. marginatus* specimens were collected in 2017 from Rumex plants growing on the CNRS campus, Gif-sur-Yvette, France (48°42'17.0"N 2°07'42.2"E) and in 2022 in the Bures – Gif-sur-Yvette water reservoir basin of Bures-sur-Yvette, France (48°41'52.4"N 2°09'09.3"E).

*R. pedestris* was reared in Petri dishes (90 mm in diameter and 20 mm height at 25°C under a long-day regimen (16 h light and 8 h dark) and fed with soybean seeds and cotton pads with distilled water containing 0.05% ascorbic acid. *C. marginatus* was reared in the same way but fed with a mixture of roasted peanut and pistachio seeds.

Bacterial strains used in this study are listed in Table S1. *Caballeronia* species were cultured in Yeast Glucose (YG) medium (0.5% yeast extract, 0.4% glucose, 0.1% NaCl) or MM medium [1], supplemented with 30 µg/mL of kanamycin (Km) or 30 µg/mL of rifampicin (Rif), if necessary, at 28°C on a gyratory shaker at 180 rpm. *Escherichia coli* strains, used for the generation of fluorescent protein tagged bacteria, were grown at 37°C in Luria-Bertani (LB) medium (5 g/L yeast extract, 10 g/L tryptone, 5 g/L NaCl), supplemented with 300 µg /mL of diaminopimelic acid (DAP), 50 µg/mL of Km, 100 µg/mL of ampicillin (Amp), or 100 µg/mL of chloramphenicol (Cm).

### Generation of fluorescent protein tagged *Caballeronia* species

GFP-labelled *Caballeronia* species were generated by inserting a mini-Tn*7* transposon containing p*lac*::*GFP* into the *attTn7* site downstream of the *glmS* gene in the chromosome of *Caballeronia* species via triparental conjugation as described previously [2]. Briefly, 200 µL overnight cultures of *Caballeronia* species were diluted into 10 mL fresh YG and incubated at 28°C at 180 rpm to an exponential phase ($OD_{600}$ = 0.5-1). These cultures were washed twice in fresh YG medium without antibiotics by centrifugation at 4,000 rpm for 10 minutes. Bacterial pellets were resuspended in fresh YG medium to obtain a final $OD_{600}$ from 5 to 10. The transposon donor strain WM3064.pURR25, the helper WM3064.pUX-BF13 were prepared in a similar way and the *Caballeronia* species, donor and helper were mixed at a 1:1:1 ratio.

The conjugation mix was spotted on YG agar plates containing DAP. After overnight incubation at 28°C, bacterial spots were resuspended in 1 mL of YG medium, diluted in ten-fold series and plated out on YG with Km 30 µg/mL. DAP was not added to counter-select the *E. coli* strains. mScarlet-I-labelled *Caballeronia* strains were generated by inserting a Tn*7* transposon encoding mScarlet-I. The plasmid pMRE-Tn7-135 [3], carrying the Tn*7* transposon with the mScarlet-I gene and the chloramphenicol resistance marker and the Tn*7* transposase genes under the control of an arabinose-inducible promoter, was introduced into the target *Caballeronia* strains by electroporation. The electroporated cells were inoculated on YG medium with 0.1% L-arabinose, which induces the expression of the plasmid-encoded transposase and incubated for 1 h at 30°C at 180 rpm. Then, the cultures were plated on YG medium supplemented with Cm and incubated at 28°C for 3 days. For GFP- and mScarlet-I-labelled *Caballeronia* strains, the brightest colonies were selected by blue LED light illumination, and purified on YG agar supplemented with antibiotics.

**Soil inoculation test in *Riptortus pedestris* and *Coreus marginatus***

Samples of soil from the CNRS campus, Gif-sur-Yvette, France (48°42'17.0"N 2°07'42.2"E) were collected in 2019 and sieved with a 2 mm sieve to remove debris. In preparation of the inoculation with the soil sample, the cotton pads with distilled water containing 0.05% ascorbic acid were removed from the petri dishes with 2$^{nd}$ instar nymphs of *R. pedestris* or *C. marginatus* and the nymphs were maintained overnight without water to make them thirsty, which facilitates the subsequent ingestion of bacteria. The 2$^{nd}$ instar is the most susceptible developmental stage for acquisition of the crypt symbionts [4]. The following day, cotton pads wetted with 1 g of soil suspension in 10 mL of water were reintroduced in the petri dishes. Throughout the experiment, the two species were housed in separate petri dishes with 10 insects per dish. *R. pedestris* and *C. marginatus* nymphs in their third instar, at five days and seven days after inoculation, respectively, were digested and their M4 crypt region was collected in 100 µL PBS (137 mM NaCl, 8.1 mM $Na_2HPO_4$, 2.7 mM KCl, and 1.5 mM $KH_2PO_4$, pH 7.5).

**Identification of gut symbionts by 16S rRNA sequencing and molecular phylogenetic analysis**

DNA was extracted from the dissected midgut crypts of individual third instar nymphs using a phenol-chloroform extraction method and a clone library analysis of the 16S rRNA gene was performed as previously reported [5]. The obtained sequences were assembled by the ATSQ software (version 5.2; Software Development, Japan), followed by manual corrections. Assembled 1500bp long sequences were associated to the most homologous bacterial species/strains by BLAST comparison. Sequences of over 99% identity were assigned to the same operational taxonomical unit (OTU).

Multiple alignment of the 16S rRNA gene was performed with MAFFT on the EMBL-EBI server [6]. A molecular phylogenetic tree was generated by the maximum likelihood (ML) method with removal of gap-including and ambiguous sites, and with bootstrap analysis (1,000 replicates) in the MEGA software (version 10.1.8) [7]. We selected the Tamura-Nei model of nucleotide substitutions with gamma distributed and invariant sites (G+I).

**Single strain infection test in *Riptortus pedestris* and *Coreus marginatus***

Two hundred µL of overnight cultures of GFP- or mScarlet-I-labelled *Caballeronia* strains were diluted into 10 mL YG medium and incubated at 28°C at 180 rpm until exponential phase ($OD_{600}$ = 0.5-1). These cultures were diluted to a final concentration of $10^7$ cfu/mL in distilled water with 0.05% ascorbic acid. The second instar nymphs of *R. pedestris* and *C. marginatus* were prepared as above, by removing drinking water, and infected by oral administration via new cotton pads wetted with the bacterial suspension. Aposymbiotic insects were obtained providing only 0.05% ascorbic acid water without bacteria. These insects were reared until reaching the third instar (5 dpi for *R. pedestris* and 7 dpi for *C. marginatus*) before dissection in PBS, under a binocular microscope and analysis of the colonization state of the midgut crypts by fluorescence microscopy observation using a Nikon, Eclipse 80i microscope (Nikon Corporation, Japan).

For fitness measurements in *R. pedestris,* insects were infected as described above with different *Caballeronia* species. Until the adult stage, dead insects were counted every day, and the day of moulting from the last larval stage into the adult stage was reported to determine development time. For morphological characterization, one day old adults were immersed in acetone. Acetone was refreshed two times and the insects were dried in free air. Each

individual was weighted and thorax width and insect total length were reported using measuring pliers. In order to calculate the effect of the symbionts on reproduction, one female and two males were placed together in a container and egg production was counted as described before [8]. Mated females begin oviposition 6 to 7 days after molting to adulthood. Upon commencement of egg-laying, the total egg count was recorded after 1 week. Statistical analysis, using a Kruskal-Wallis test, Dunn post hoc test and Benjamini-Hochberg correction, was performed with R studio (version 4.5.0) [9].

**Two strain competition assays in *Riptortus pedestris* and *Coreus marginatus***

In each pairwise competition assay, we combined GFP- and mScarlet-I-labelled *Caballeronia* strains (Table S1). Exponential phase cultures of each strain were diluted to a final concentration of $10^7$ cfu/mL in distilled water with 0.05% ascorbic acid and combined in equal amounts. The composition of the mixture of the two strains was verified by flow cytometry analysis as described below and corrections in the mixture were introduced until reaching a 1:1 mixture. Inoculation of *R. pedestris* and *C. marginatus* insects with the mixed inoculum was performed as described above. These insects were maintained until the third instar nymphs.

Symbiotic organs were dissected under a binocular microscope in sterilized PBS. In part of the samples, a qualitative estimation of the colonization state of the midgut crypts by the GFP- and mScarlet-I-labelled strains was determined by fluorescence microscopy using a Nikon, Eclipse 80i microscope (Nikon Corporation, Japan). In the remaining samples, each M4 was individually grounded by a sterile pestle in a 1.5 mL tube containing 100 µL of sterilized PBS. The Pestle was washed with 400 µL of PBS and these samples were stored at 4°C for a maximum of 5 days until flow cytometry analysis. The relative number of GFP- and mScarlet-I-tagged bacterial strains in each sample was analysed by flow cytometry using a CytoFlex S machine (Beckman Coulter, US). GFP signal was excited by a 488 nm laser and a 525/40 nm band pass filter was used for detection; mScarlet-I signal was excited by a 561 nm laser and a 610/20 nm band pass filter was used for detection. In data acquisition of flow cytometry analysis using the Cytexpert software (version 2.4), a first gating for bacterial cells was made on the forward-scatter (FSC)-side scatter (SSC) dot plot to focus on bacteria, and then doublets were discarded using the SSC_Area and SSC_Height dot plot. Data acquisition for a total of

50,000-100,000 bacteria was recorded for each condition. Thresholds for considering positive GFP and mScarlet-I bacteria were determined using cultures of non-fluorescent bacteria as a negative control and cultures of the corresponding tagged strains as positive controls. A competition index (CI) was calculated as the ratio of a tested GFP-strain to a mScarlet-I-strain, normalized by the ratio of the inoculum, which was always close to 1. Statistical analysis, using a Kruskal-Wallis test, Dunn post hoc test and Benjamini-Hochberg correction, was performed with R studio (version 4.5.0) [9].

**Motility and chemotaxis measurements**

To measure the velocity of the different strains in liquid YG medium, time-lapse videos of the bacteria's movement were made using Leica DMI6000 B Inverted Research Microscope (Leica Camera AG, Germany). Cultures of the GFP-labelled strains in exponential phase ($OD_{600}$<1), grown in YG medium, were diluted to $OD_{600}$ = 0.05, and loaded on a glass slide inside a Frame-Seal (Bio-Rad Laboratories, Inc, US) barricade, then covered with a piece of Polydimethylsiloxane (PDMS) to allow the entry of oxygen during microscopy. For the imaging, the YFP fluorescence filter and 10X objective was used. Videos of 10 seconds at a frequency of 10 frames per second were recorded. The tracking and analysis of the motility was done using the TrackMate plugin [10] of the Fiji software (version 2.9.0) [11], which identifies the movement of a fluorescent particle (bacteria) from frame to frame, forms tracks of individual particles across all the frames and extract the coordinates of the particles along the tracks. From the coordinates and time difference between frames, instantaneous velocities are calculated. For each strain, from 500-1,500 tracks about 50,000 to 150,000 instant velocities were calculated, which were represented in box plots using R Studio (R version 4.5.0) [9].

To perform the chemotaxis measurements, swimming plates (YG medium with 0.3% agar) were inoculated by inserting into the soft agar layer 2 µL of a suspension of the tested strains, grown in exponential phase ($OD_{600}$ < 1) and concentrated to $OD_{600}$ = 2. The plates were incubated at 28°C in a damp environment to make sure the plates did not dry out. Images of the plates were taken at regular time points for around 60 hours, and the diameter of growth in the plate was measured on the pictures using Fiji.

**Two strain competition assays in liquid culture and on agar plates**

For the pairwise competition experiments, a GFP-tagged and an mScarlet-tagged strain were mixed together in a 1:1 ratio, verified by flow cytometry, and adjusted as above. The strain mixtures were then inoculated either in liquid cultures with MM medium and either glucose or succinate as carbon source (inoculum at $OD_{600}$ = 0.05), on standard (1.5% agar) YG plates (spot inoculum of 50 µL at $OD_{600}$ = 0.05 or $OD_{600}$ = 2; the liquid of the spots was air dried to create inter-strain cell-cell contact on the agar plates during colony growth), or on swimming plates as above (YG medium with 0.3% agar; inoculum at $OD_{600}$ = 2). Co-cultures were grown for 24 hours at 28°C. The cells were harvested in 1X PBS buffer from across the growth for liquid cultures and solid agar plates or from the edges only of the growth zone for the swimming plates. The relative number of GFP- and mScarlet-I-labelled bacteria in the harvested cells of each sample was measured by flow cytometry as above.

**Biolog phenotype microarrays**

The metabolic profile of strains was analysed using four 96-well Phenotype Microarray (Biolog Inc., US] microplates, which consisted of two microplates of carbon substrates (PM1 and PM2A), one microplate of various nitrogen substrates (PM3B) and one microplate of different phosphorus and sulfur substrates (PM4A). The principle of the assay is based on the measurement of purple color formation from the irreversible redox reaction of the Biolog tetrazolium dye, in response to the production of reduced nicotinamide adenine dinucleotide (NADH) by bacterial respiration, which is active when the bacteria are able to uptake and metabolize the test compound present in the well. The assay was performed according to the manufacturer's protocol for Gram-negative bacteria. For the analysis, single colonies of the strains were harvested from a freshly prepared YG agar plate and suspended in 1X IF-0 solution (Biolog Inc., US). That suspension was further diluted using IF-0+Dye Mix A solution (Biolog Inc., US) to attain $OD_{600nm} \approx 0.07$. The prepared bacterial suspension was then added to each of the wells of the PM1 and PM2A plates. For the plates lacking carbon sources (PM3B and PM4A), the pre-prepared 100X carbon stock solution (2 M glucose, 200 µM ferric citrate) was added additionally in the IF-0+Dye Mix A solution for the preparation of the sample. The plates were incubated at 28°C in a humidified box. Kinetic absorbance data at $OD_{490nm}$ and $OD_{750nm}$ for each well were obtained at regular time intervals during 62h using a Spark

Multimode Microplate Reader (Tecan, Switzerland). A corrected signal was calculated by subtracting the $OD_{750nm}$ measurement from the $OD_{490nm}$ measurement and the area under the kinetic curve was calculated in Excel (Microsoft Corporation, US). Values were normalized per plate and the full data set for the 4 arrays and all strains was analyzed and visualized in a heat map by MEV software (version 4.8.0) [12] using the hierarchical clustering function with Pearson Correlation distance metric and average linkage settings.

**Sensitivity assays to antimicrobial peptides**

Precultures of tested strains were grown in MM medium with glucose as carbon source. Overnight grown cultures were diluted to an $OD_{600nm} = 0.3$ in fresh medium and grown until they reached $OD_{600nm} \approx 1$. The cells were pelleted by centrifugation, resuspended in fresh medium and diluted to $OD_{600nm} = 0.05$. These cell suspensions were dispatched in a 96-well plate at 146.25 µL per well and 3.75 µL AMPs [13], dissolved in water at a 40-fold concentration, were added and mixed with the bacterial suspension by pipetting up and down. The AMPs were tested at the following concentrations (all in µg/mL): Polymyxin B (PMB) at 50, 25, 12.5, 6.25, 3.125, 1.5625, 0.78; riptocin, thanatin, CCR0179, CCR0191, CCR0776, CCR1659 at 300, 250, 125, 50; LL37 at 40, 25, 12.5, 5; NCR335 at 100, 50, 25, 12.5. The 96-well plates were incubated in a SPECTROstar Nano plate incubator (BMG Labtech GmbH, Germany). The growth of the cultures in the wells was monitored by measuring the $OD_{600nm}$ and data points were collected every hour for 48 h. Plates were incubated at 28°C with double orbital shaking at 200 rpm. Data and growth curves were analyzed using Microsoft Excel (Microsoft Corporation, US). The minimal concentration was determined at which growth was diminished compared to the untreated control. The assays were performed in biological triplicates for all peptides.

**Nucleotide sequence accession numbers**

The nucleotide sequence data of the 16S rRNA gene obtained in the present study have been deposited in the DDBJ public database with the accession numbers LC816739-LC817101.

## References


1. Jouan R, Lextrait G, Lachat J *et al.* Transposon sequencing reveals the essential gene set and genes enabling gut symbiosis in the insect symbiont *Caballeronia insecticola*. *ISME Commun* 2024;**4**:ycad001. https://doi.org/10.1093/ismeco/ycad001
2. Kikuchi Y, Fukatsu T. Live imaging of symbiosis: spatiotemporal infection dynamics of a GFP-labelled *Burkholderia* symbiont in the bean bug *Riptortus pedestris*. *Mol Ecol* 2014;**23**:1445-56. https://doi.org/10.1111/mec.12479
3. Schlechter RO, Jun H, Bernach M *et al.* Chromatic bacteria - A broad host-range plasmid and chromosomal insertion toolbox for fluorescent protein expression in bacteria. *Front Microbiol* 2018;**9**:3052. https://doi.org/10.3389/fmicb.2018.03052
4. Kikuchi Y, Hosokawa T, Fukatsu T. Specific developmental window for establishment of an insect-microbe gut symbiosis. *Appl Environ Microbiol* 2011;**77**:4075-81. https://doi.org/10.1128/AEM.00358-11
5. Ohbayashi T, Itoh H, Lachat J *et al. Burkholderia* gut symbionts associated with European and Japanese populations of the dock bug *Coreus marginatus* (Coreoidea: Coreidae). *Microbes Environ* 2019;**34**:219-22. https://doi.org/10.1264/jsme2.ME19011
6. Li W, Cowley A, Uludag M *et al.* The EMBL-EBI bioinformatics web and programmatic tools framework. *Nucleic Acids Res* 2015;**43(W1)**:W580-4. https://doi.org/10.1093/nar/gkv279
7. Kumar S, Stecher G, Li M *et al.* MEGA X: Molecular Evolutionary Genetics Analysis across Computing Platforms. *Mol Biol Evol* 2018;**35**:1547-9. https://doi.org/10.1093/molbev/msy096
8. Jang S, Ishigami K, Mergaert P, Kikuchi Y. Ingested soil bacteria breach gut epithelia and prime systemic immunity in an insect. *Proc Natl Acad Sci U S A* 2024;**121**:e2315540121. https://doi.org/10.1073/pnas.2315540121
9. R Core Team. R: A language and environment for statistical computing. R Foundation for Statistical Computing, Vienna, Austria. 2021. https://www.R-project.org/
10. Ershov D, Phan MS, Pylvänäinen JW *et al.* TrackMate 7: integrating state-of-the-art segmentation algorithms into tracking pipelines. *Nat Methods* 2022;**19**:829-32. https://doi.org/10.1038/s41592-022-01507-1
11. Schindelin J, Arganda-Carreras I, Frise E *et al.* Fiji: an open-source platform for biological-image analysis. *Nat Methods* 2012;**9**:676-82. https://doi.org/10.1038/nmeth.2019
12. Saeed AI, Sharov V, White J *et al.* TM4: a free, open-source system for microarray data management and analysis. *Biotechniques* 2003;**34**:374-8. https://doi.org/10.2144/03342mt01
13. Lachat J, Lextrait G, Jouan R *et al.* Hundreds of antimicrobial peptides create a selective barrier for insect gut symbionts. *Proc Natl Acad Sci U S A* 2024;**121**:e2401802121. https://doi.org/10.1073/pnas.2401802121
